# Association of Maternal Cardiac Arrhythmias with Pregnancy Outcomes: A Systematic Review and Meta-Analysis

**DOI:** 10.3390/healthcare14080993

**Published:** 2026-04-09

**Authors:** Antonios Siargkas, Alexandra Arvanitaki, Areti Faka, Efstratios Karagiannidis, Barbara Fyntanidou, Apostolos Mamopoulos, Antonios P. Antoniadis, Nikolaos Fragakis, Themistoklis Dagklis, Ioannis Tsakiridis

**Affiliations:** 1 3rd Department of Obstetrics and Gynecology, School of Medicine, Faculty of Health Sciences, Aristotle University of Thessaloniki, 54124 Thessaloniki, Greece; asiargk@auth.gr (A.S.); aretifaka@auth.gr (A.F.); amamop@auth.gr (A.M.); dagklis@auth.gr (T.D.); 2 2nd Cardiology Department, School of Medicine, Faculty of Health Sciences, Aristotle University of Thessaloniki, 54124 Thessaloniki, Greece; aarvanic@auth.gr (A.A.); aantoniadis@auth.gr (A.P.A.); nfrag@auth.gr (N.F.); 3 Department of Emergency Medicine, School of Medicine, Faculty of Health Sciences, Aristotle University of Thessaloniki, 54124 Thessaloniki, Greece; ekaragij@auth.gr (E.K.); fyntanidou@auth.gr (B.F.)

**Keywords:** maternal cardiac arrhythmias, pregnancy outcomes, preeclampsia, ventricular tachycardia, supraventricular tachycardia, stillbirth, preterm birth, cardio-obstetrics, meta-analysis

## Abstract

**Highlights:**

**What are the main findings?**

- The presence of any maternal cardiac arrhythmia is significantly associated with an increased overall risk for adverse pregnancy outcomes, including preeclampsia, placental abruption, cesarean section, preterm delivery, and small-for-gestational-age neonates.
- Ventricular tachyarrhythmias are strongly associated with severe perinatal mortality, including a four-fold increase in stillbirth and a significant rise in neonatal deaths reflecting acute hemodynamic compromise, whereas supraventricular tachycardia is independently linked to signs of chronic placental insufficiency, including preeclampsia, preterm delivery, and small-for-gestational-age neonates.

**What are the implications of the main findings?**

- The onset of a cardiac arrhythmia during pregnancy may serve as a sentinel marker for underlying vascular dysfunction, warranting enhanced monitoring for preeclampsia and fetal growth restriction.
- Given the high risk of neonatal morbidity and mortality associated with sustained tachyarrhythmias, management should ideally occur at centers equipped with advanced neonatal intensive care capabilities.

**Abstract:**

**Introduction:** The prevalence of maternal arrhythmias is increasing with advanced maternal age. Current evidence regarding the association between maternal arrhythmias and pregnancy outcomes remains inconsistent. This meta-analysis aimed to define the associations between adverse pregnancy outcomes and specific maternal arrhythmias. **Methods:** We conducted a systematic review and meta-analysis using the PRISMA guidelines. We searched MEDLINE, Scopus, and Cochrane on the 4th of November 2025 for cohort and case–control studies comparing pregnant women with cardiac arrhythmias to those without. Primary outcomes included preeclampsia, stillbirth, preterm delivery, and small-for-gestational-age (SGA) neonates. Data were pooled using random-effects models with subgroup analyses by arrhythmia type. **Results:** Nineteen studies were included. Maternal arrhythmias were associated with a significantly increased risk of preeclampsia (RR = 1.46, 95% CI [1.10, 1.93]), preterm delivery (RR = 1.39, 95% CI [1.12, 1.72]), and stillbirth (RR = 2.09, 95% CI [1.11, 3.91]). Ventricular tachycardia/fibrillation was linked to the most severe outcomes, including a four-fold increase in stillbirth (RR = 4.20, 95% CI [3.75, 4.71]) and a fifteen-fold increase in neonatal death (RR = 15.47, 95% CI [3.45, 69.45]). Supraventricular tachycardia was independently associated with preeclampsia (aRR = 1.14, 95% CI [1.04, 1.24]), preterm delivery (aRR = 1.76, 95% CI [1.39, 2.23]), and SGA neonates (aRR = 5.93, 95% CI [1.23, 28.55]). Risks were notably higher in the general population compared to women with known heart disease, supporting an “unmasking” of occult vulnerability. **Conclusions:** Maternal arrhythmias are associated with distinct fetal risks beyond maternal hemodynamics. Ventricular tachycardia was associated with severe outcomes, likely reflecting acute compromise, while supraventricular tachycardia was linked to signs of chronic vascular dysfunction. These findings suggest arrhythmias as possible sentinels for placental insufficiency, necessitating enhanced surveillance.

## 1. Introduction

Cardiovascular disease has been established as a major contributor to preeclampsia and other hypertensive disorders of pregnancy in the US, with national data confirming a sharp increase in the prevalence of cardiovascular conditions among pregnant women, rising from 9.2% in 2010 to 14.8% by 2019 [1,2]. Cardiac arrhythmias are a major manifestation of cardiovascular burden in pregnancy, with an increasing trend, primarily driven by critical demographic and clinical shifts, like the improved survival of women with congenital heart disease to reproductive age and the increasing tendency to delay motherhood, which is associated with a rise in cardiovascular comorbidities [3].

Pregnancy inherently imposes a pro-arrhythmic state, driven by profound hemodynamic adaptations—such as a 50% increase in plasma volume and elevated cardiac output—alongside hormonal fluctuations that heighten adrenergic sensitivity [4]. Against this backdrop of physiological stress, preconception health plays a pivotal role in determining individual susceptibility. Maternal characteristics such as advanced age, obesity, and lifestyle factors, including tobacco and alcohol use, do not merely predispose women to long-term cardiovascular disease; they may also create a vulnerable substrate by promoting subclinical vascular dysfunction or structural remodeling prior to conception [5,6]. Consequently, the hemodynamic burden of gestation acts as a cardiovascular ‘stress test,’ interacting with these pre-existing risk factors to potentially unmask latent arrhythmic tendencies, while simultaneously serving as independent drivers for the adverse obstetrical outcomes under investigation [5].

Due to these physiological shifts, the cardiac arrhythmias encountered during pregnancy are diverse, with atrial fibrillation (AF) currently ranking as the most prevalent, followed in frequency by supraventricular tachycardia (SVT) and ventricular tachycardia (VT), alongside benign entities such as premature ventricular contractions (PVCs) [7]. Beyond maternal symptoms, these rhythm disturbances may have the potential to compromise uteroplacental perfusion, potentially posing risks to fetal growth and survival. However, existing evidence regarding the magnitude and specificity of these risks remains fragmented [8]. Management is further complicated by therapeutic constraints, as pharmacological and interventional options are limited by fetal safety considerations. Concerns regarding teratogenicity in the first trimester and fetal radiation exposure during catheter ablation necessitate a coordinated, multidisciplinary approach [9,10]. Given the complexity and the inherent dual risk to both the mother and the fetus, managing these arrhythmias necessitates a coordinated, multidisciplinary approach involving cardiology, electrophysiology and maternal–fetal medicine specialists [9,11].

This management complexity is further exacerbated by a scattered and methodologically inconsistent body of research, which has yet to establish definitive risk estimates for pregnancy outcomes. Divergent findings on preeclampsia illustrate this evidentiary discord: in pregnant women with SVT, reported associations range from a pronounced 25% elevation in risk [12] to a subtle, possibly protective effect [13]. This inconsistency also encompasses the most severe perinatal outcomes, such as stillbirth; the association between VT and stillbirth remains equally contentious, with published estimates ranging from no discernible effect [14] to more than a fourfold increase in risk [15].

This meta-analysis aims to comprehensively evaluate and integrate the existing evidence to define more accurate and data-driven associations for adverse pregnancy outcomes with specific maternal cardiac arrhythmias.

## 2. Data Synthesis and Methods

### 2.1. Study Oversight and Protocol Registration

This systematic approach was governed by the Preferred Reporting Items for Systematic reviews and Meta-Analyses (PRISMA) statement [16]. Before initiating our investigation, we formally documented a comprehensive protocol, which detailed our analytical strategy and aims, in the Open Science Framework (10.17605/OSF.IO/K6B4X). Since this project solely involved the synthesis of previously published material, no institutional review board was needed.

### 2.2. Literature Search and Identification Strategy

A comprehensive literature review was undertaken to identify relevant studies, structured around the Population, Intervention, Comparison, and Outcome (PICO) framework. Major databases, including MEDLINE, Scopus, and The Cochrane Library, were thoroughly examined using a combination of keywords and controlled vocabulary pertaining to cardiac arrhythmias and adverse pregnancy outcomes (Supplementary Document S1). Duplicate records were meticulously removed using reference management software (Rayyan [17]). Two independent reviewers (A.S. and A.F.) conducted an iterative, two-stage screening process—initially evaluating titles and abstracts, followed by a detailed full-text review against predefined inclusion criteria. Any discrepancies during screening were resolved by a third reviewer (A.A.). Additionally, manual searches of reference lists from all included articles were performed to ensure comprehensiveness of the search.

### 2.3. Eligibility Criteria and Study Population

We defined precise eligibility criteria. Original studies qualified if they used a cohort or case–control design and examined the association between maternal cardiac arrhythmias and pregnancy complications. The focus population was pregnant women, and we defined exposure as any diagnosis of a maternal cardiac rhythm abnormality, such as AF, atrial flutter (AFl), SVT, VT, ventricular fibrillation (VF), or pre-excitation syndromes. The comparison group consisted of pregnant women who did not have the specific arrhythmia under examined. Our team excluded publications such as case reports and conference abstracts, along with studies that provided insufficient raw data for our subsequent meta-analysis.

### 2.4. Endpoints and Outcome Classification

Our primary metrics encompassed preeclampsia, stillbirth, preterm delivery, and neonates identified as small-for-gestational-age (SGA). In addition, we examined several secondary outcomes, which included cesarean section, gestational hypertension and gestational diabetes mellitus (GDM) on the maternal side and neonatal death, neonatal intensive care unit (NICU) admission, and a 5 min Apgar score < 7 on the offspring side.

### 2.5. Data Extraction and Quality Assurance

We developed a specialized abstraction tool using Microsoft Excel to standardize information capture. Two investigators (A.S. and A.F.) individually collected critical information from each selected study. This entailed the compilation of details regarding study design and demographics; clinical facts about the patient cohort, arrhythmia type, and treatment protocols; and the quantitative data for the investigated outcomes. For dichotomous results, we tabulated the number of incidents and the total sample size to derive RRs, prioritizing multivariable-adjusted effect estimates and their 95% CIs when available.

Two independent reviewers (A.S. and A.F.) evaluated the methodological integrity of the included articles in duplicate. We employed the Newcastle–Ottawa Scale (NOS) to appraise the quality of the observational studies based on criteria for selection, comparability, and outcome ascertainment. Concurrently, we utilized the Quality In Prognosis Studies (QUIPS) tool to systematically assess the risk of bias across six key domains, including study confounding and outcome measurement. A third team member (A.A.) arbitrated any divergences in assessment between the two reviewers.

### 2.6. Statistical Synthesis and Robustness Evaluation

We conducted a formal quantitative synthesis for each outcome reported by at least three studies. We chose the RR with its corresponding 95% CI as the summary effect measure, and we derived it from the raw event data. Heterogeneity arising from the observational design of the included studies was anticipated; therefore, the data were pooled using a random-effects model according to the DerSimonian and Laird method. Detailed subgroup analyses were subsequently conducted to identify and explain potential sources of heterogeneity, and adjusted analyses were performed to account for possible confounding. We assessed for statistical heterogeneity using the Cochran’s Q test and measured its extent with the I^2^ statistic. When a meta-analysis included 10 or more studies, we investigated potential publication bias through visual inspection of funnel plots and the formal Egger’s test.

To explore sources of heterogeneity and test the stability of our results, we performed several planned secondary analyses. We conducted subgroup analyses by stratifying results based on the specific arrhythmia type (AF/AFl, SVT, VT/VF, and a mixed arrhythmia cohort comprising studies that did not report outcomes separately for specific arrhythmia subtypes) and, where data were provided, by the presence of underlying structural heart disease (categorized into women with known cardiac disease, women without known cardiac disease, while women that did not belong in any of these groups and were all comers either as outpatients or inpatients were categorized as “general obstetric population”).

Meta-regression was not performed due to the limited number of studies per subgroup (<10) and the lack of consistent reporting of granular covariate data (e.g., specific BMI values or smoking rates) across the included studies. We performed an adjustment analysis by pooling multivariable-adjusted risk ratios (aRRs) when at least three studies provided them. We ultimately did not perform an additional sensitivity analysis, in which we had planned to exclude studies with a high risk of bias, because our analysis of adjusted estimates addressed the primary driver of that risk.

## 3. Results

### 3.1. Selection of Included Studies

**Figure 1:**
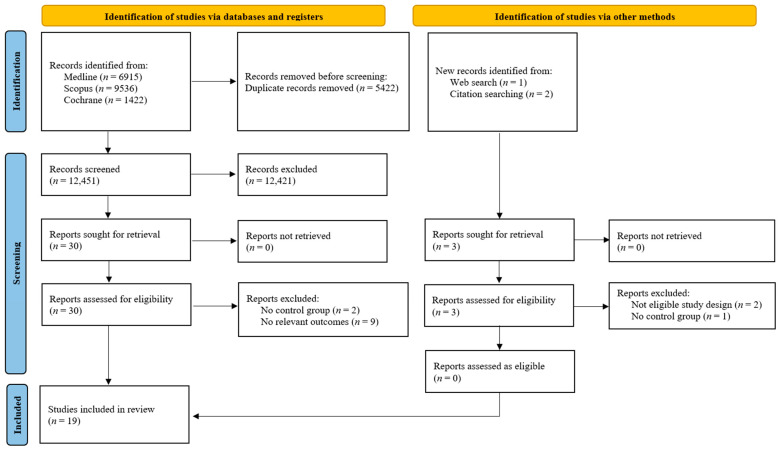
Study selection flowchart.

Our comprehensive query, conducted on the 4th of November 2025 across three electronic databases (Medline, Scopus, and Cochrane), identified 17,873 total records. Following the removal of 5422 duplicates, 12,451 unique articles proceeded to the initial screening phase. During this phase, 12,421 records were excluded, leaving 30 articles from the database search. An additional 3 records were identified through other methods, bringing the total number of articles for full-text assessment to 33. After a detailed review of these full texts, we excluded 14 studies for reasons that included an ineligible design (*n* = 2), the absence of a control group (*n* = 3), or failure to report relevant outcomes (*n* = 9). Ultimately, we included 19 studies in the final systematic review and meta-analysis [12,13,14,15,18,19,20,21,22,23,24,25,26,27,28,29,30,31,32]. Figure 1 illustrates the full selection process.

### 3.2. Body of Included Evidence

The evidence encompassed a broad spectrum of arrhythmias, including AF/AFl, SVT, VT/VF, and PVCs. Most investigations applied statistical methods, most commonly multivariable regression, to adjust for potential confounders. The included studies were primarily retrospective cohort analyses, with some prospective cohort and case–control designs also represented. Considerable variation existed among the patient populations: some studies focused on pregnant women with structurally normal hearts, whereas others examined cohorts with pre-existing cardiac conditions, such as congenital or rheumatic heart disease. To address population heterogeneity, all available data on potential confounders were systematically extracted and categorized, including antiarrhythmic therapy, anticoagulation use, ablation history, specific structural heart diseases (e.g., rheumatic mitral valve disease and congenital anomalies), arrhythmia duration, and the adequacy of rate or rhythm control. These detailed clinical characteristics, together with study demographics (Table 1), are presented in Supplementary Table S1 to facilitate contextual interpretation of the findings. However, inconsistent reporting across the primary studies precluded their incorporation into a quantitative meta-regression or additional subgroup analyses.

### 3.3. Quality of the Included Studies

All 19 included studies were judged to be of high methodological quality according to the NOS, with total scores consistently ranging from 7 to 9 on the 9-point scale. Specifically, eight studies attained a score of 9 [12,15,18,19,20,21,23,29], five achieved a score of 8 [13,14,28,31,32], and six received 7 points [22,24,25,26,27,30]. The most frequent area of potential limitation was identified in the “Comparability” domain: six studies did not obtain a star in this category, suggesting that their design or analysis may not have adequately adjusted for key confounding variables. Nevertheless, the included literature performed strongly in the NOS domains assessing cohort selection and outcome ascertainment, reflecting well-defined case criteria and reliable data collection procedures (Table 2).

### 3.4. Pregnancy Outcomes

#### 3.4.1. Preeclampsia

**Figure 2:**
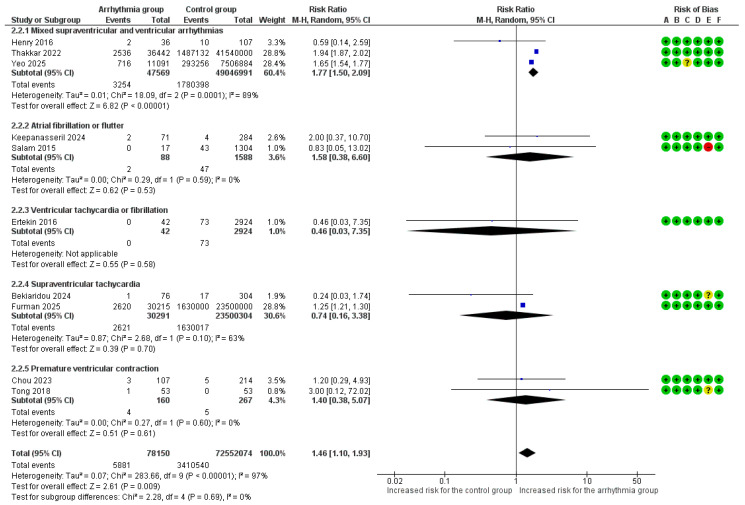
Forest plot of the association between maternal cardiac arrhythmias and preeclampsia (using a random-effects model). Abbreviations: CI, Confidence Interval; M–H, Mantel–Haenszel method [12,13,14,15,20,21,23,27,29,30].

Ten studies provided raw data and were included in this analysis [12,13,14,15,20,21,23,27,29,30]. The overall risk of preeclampsia was significantly increased by 46% in pregnant women with any arrhythmia (RR = 1.46, 95% CI [1.10, 1.93]), an analysis characterized by substantial heterogeneity (I^2^ = 97%). The test for arrhythmia subgroup differences demonstrated that there was no significant variation in the risk of preeclampsia between the different arrhythmia types (*p* = 0.69) (Figure 2). A sensitivity analysis for preeclampsia using adjusted estimates found a significant 14% increase in the overall risk (aRR = 1.14, 95%CI [1.04, 1.25]). This association was primarily driven by SVT, which showed a similarly significant increase in risk (aRR = 1.14, 95%CI [1.04, 1.24]). The analyses for AF/AFl and PVCs did not show a significant association after adjustment (Supplementary Figure S1). A subgroup analysis for preeclampsia based on underlying heart disease found that a significant 59% increased risk was linked to arrhythmias occurring in the general population (RR = 1.59, 95%CI [1.17, 2.16]). No significant association was found in the subgroups of women with or without known cardiac disease, and there was no significant difference in risk between the three populations (*p* = 0.27) (Supplementary Figure S2).

#### 3.4.2. Hypertensive Disorders of Pregnancy

Six studies provided raw data and were included in this analysis [14,15,20,23,27,30]. The overall risk for hypertensive disorders of pregnancy was significantly increased by 25% in women with any type of arrhythmia (RR = 1.25, 95%CI [1.17, 1.33]), with no heterogeneity observed across the studies (I^2^ = 0%). In the subgroup analyses, a significant association was only found in the mixed arrhythmias cohort (RR = 1.25, 95%CI [1.17, 1.33]). The test for subgroup differences confirmed no significant variation in risk between the different arrhythmia types (*p* = 0.53) (Supplementary Figure S3). A sensitivity analysis using adjusted estimates found no significant association between any arrhythmia and hypertensive disorders of pregnancy (aRR = 1.03, 95%CI [0.59, 1.78]). Similarly, no significant risk was found in the adjusted analyses by different arrhythmia subgroups (Supplementary Figure S4).

#### 3.4.3. Gestational Diabetes Mellitus

Six studies provided raw data and were included in this analysis [15,20,21,23,29,31]. The overall risk of GDM was not significantly increased in pregnant women with any arrhythmia (RR = 1.42, 95%CI [0.94, 2.16]), an analysis characterized by substantial heterogeneity (I^2^ = 98%). In the subgroup analyses, only SVT was associated with a significantly increased risk (RR = 1.81, 95%CI [1.16, 2.84]). There was no significant variation in the risk of GDM between the different arrhythmia types (*p* = 0.23) (Supplementary Figure S5). In a sensitivity analysis using adjusted estimates, the risk of GDM was not significantly associated with any arrhythmia (aRR = 1.05, 95%CI [0.76, 1.44]). The adjusted analyses for arrhythmia subtypes also showed no significant associations (Supplementary Figure S6).

#### 3.4.4. Cesarean Section

Nine studies provided raw data and were included in this analysis [13,14,20,23,27,28,29,31,32]. The overall risk of CS was significantly increased by 27% in pregnant women with any type of arrhythmia (RR = 1.27, 95%CI [1.13, 1.42]), an analysis with substantial heterogeneity (I^2^ = 70%). The subgroup analyses revealed a significantly increased risk of CS in women with VT/VF (RR = 1.47, 95%CI [1.20, 1.81]) and SVT (RR = 1.38, 95%CI [1.23, 1.56]). In contrast, PVCs were associated with a significant 25% decreased risk of CS (RR = 0.75, 95%CI [0.59, 0.97]), while AF/AFl showed no significant association. The risk varied significantly across the different arrhythmia types (*p* = 0.0002) (Supplementary Figure S7). In the sensitivity analysis for CS using adjusted estimates, the overall risk was not significantly altered (aRR = 1.10, 95% CI [0.68, 1.79]). However, the adjusted analysis revealed a significant 62% increased risk for CS in women with SVT (aRR = 1.62, 95%CI [1.45, 1.80]) and a 43% decreased risk for PVCs (aRR = 0.57, 95%CI [0.36, 0.91]). No significant associations were found for AF/AFl or VT/VF after adjustment (Supplementary Figure S8). In a subgroup analysis for the risk of CS based on the underlying heart disease, a significantly increased risk was found in both the general population (RR = 1.36, 95%CI [1.34, 1.38]) and in women with a known cardiac disease (RR = 1.31, 95% CI [1.14, 1.51]). No significant association was found in the subgroup of women without a prior cardiac diagnosis, and there was no significant difference in risk between the three populations (*p* = 0.71) (Supplementary Figure S9).

#### 3.4.5. Placental Abruption

Four studies provided raw data and were included in this analysis [20,21,29,31]. The presence of any arrhythmia was associated with a significant 2.6-fold increased risk of placental abruption (RR = 2.59, 95%CI [2.29, 2.93]), with no heterogeneity observed across studies (I^2^ = 0%). In the subgroup analyses, a statistically significant increase in risk was found in the mixed arrhythmias cohort (RR = 3.11, 95%CI [1.08, 8.97]). There was no significant variation in the risk of placental abruption between the different arrhythmia types (*p* = 0.75) (Supplementary Figure S10).

#### 3.4.6. Stillbirth

**Figure 3:**
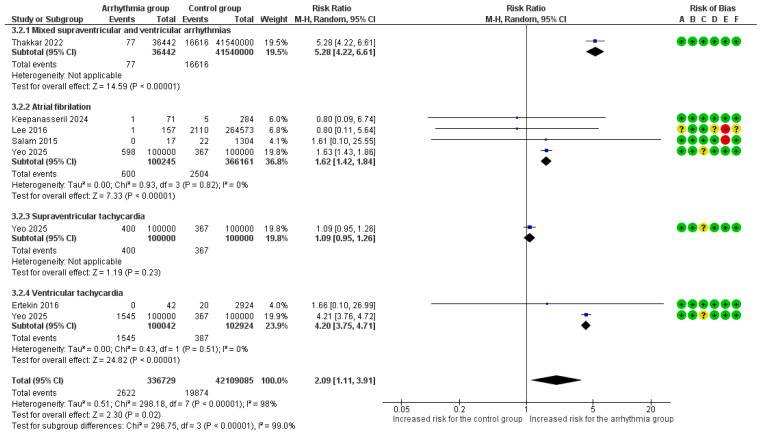
Forest plot of the association between maternal cardiac arrhythmias and stillbirth (using a random-effects model). Abbreviations: CI, Confidence Interval; M–H, Mantel–Haenszel method [14,15,23,24,27,29].

Six studies provided raw data and were included in this analysis [14,15,23,24,27,29]. The overall risk of stillbirth was doubled in pregnancies complicated by any arrhythmia subtype (RR = 2.09, 95%CI [1.11, 3.91]), although the analysis exhibited substantial heterogeneity (I^2^ = 98%). Subgroup analyses revealed significant variations in the risk of stillbirth by arrhythmia subtype (*p* < 0.001). The risk of stillbirth was significantly increased in women with VT/VF (RR = 4.20, 95%CI [3.75, 4.71]) and AF/AFl (RR = 1.62, 95%CI [1.42, 1.84]). In contrast, SVT was not associated with a significant increase in risk (Figure 3). In the sensitivity analysis for stillbirth using adjusted estimates, the overall risk was not significantly altered (aRR = 1.31, 95%CI [0.86, 2.00]). However, the adjusted analysis revealed an increased risk for stillbirth in pregnant women with VT/VF (aRR = 3.83, 95%CI [1.21, 12.12]). No significant associations were found after adjustment in AF/AFl or SVT subgroups (Supplementary Figure S11). In a subgroup analysis for stillbirth based on the underlying heart disease, no significant association was found in either the general population (RR = 2.20, 95%CI [0.69, 7.01]) or in women with a known cardiac disease (RR = 1.18, 95%CI [0.28, 4.99]) (*p* = 0.51) (Supplementary Figure S12).

#### 3.4.7. Preterm Delivery

**Figure 4:**
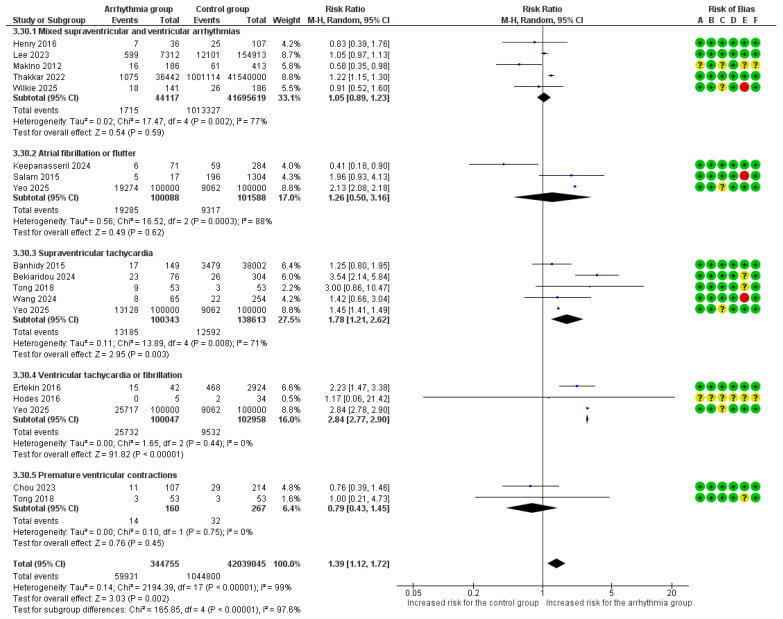
Forest plot of the association between maternal cardiac arrhythmias and preterm delivery (using a random-effects model). Abbreviations: CI, Confidence Interval; M–H, Mantel–Haenszel method [13,14,15,18,20,21,22,23,24,26,27,29,30,31,32].

Fifteen studies provided raw data and were included in this analysis [13,14,15,18,20,21,22,23,25,26,27,29,30,31,32]. The overall risk of preterm delivery was significantly increased by 39% in women with any arrhythmia (RR = 1.39, 95%CI [1.12, 1.72]), an analysis characterized by extreme heterogeneity (I^2^ = 99%). Subgroup analyses revealed significant variations by arrhythmia type. A significantly increased risk was observed for VT/VF (RR = 2.84, 95%CI [2.77, 2.90]) and SVT (RR = 1.78, 95%CI [1.21, 2.62]). In contrast, the analyses for a mixed arrhythmia cohort, AF/AFl, and PVCs did not yield statistically significant results. The test for subgroup differences confirmed that the risk for preterm delivery varied significantly across the different arrhythmia types (*p* < 0.00001) (Figure 4). In the sensitivity analysis for preterm delivery using adjusted estimates, the overall risk was significantly increased (aRR = 1.73, 95%CI [1.30, 2.30]). The adjusted analysis revealed that this association was driven by SVT, which showed a significant 76% increased risk (aRR = 1.76, 95%CI [1.39, 2.23]). No significant associations were found for AF/AFl, VT/VF, or PVCs after adjustment (Supplementary Figure S13).

#### 3.4.8. Small-for-Gestational-Age Neonates

**Figure 5:**
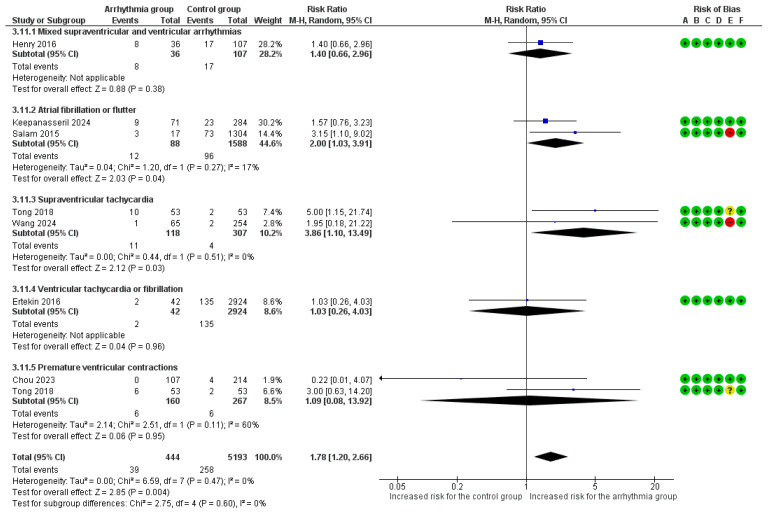
Forest plot of the association between maternal cardiac arrhythmias and small for gestational age (using a random-effects model). Abbreviations: CI, Confidence Interval; M–H, Mantel–Haenszel method [14,20,21,23,27,30,31].

Seven studies provided raw data and were included in this analysis [14,20,21,23,27,30,31]. The overall risk for delivering SGA neonates was significantly increased by 78% in pregnancies complicated by any type of arrhythmia (RR = 1.78, 95%CI [1.20, 2.66]), with low heterogeneity across studies (I^2^ = 0%). In the subgroup analyses, a significantly increased risk was observed for AF/AFl (RR = 2.00, 95%CI [1.03, 3.91]) and SVT (RR = 3.86, 95%CI [1.10, 13.49]). However, there was no significant variation in risk among various arrhythmia subgroups (*p* = 0.60) (Figure 5). In the sensitivity analysis for SGA using adjusted estimates, the overall risk was significantly increased (aRR = 2.16, 95%CI [0.86, 5.46]). The adjusted analysis revealed a significant increase in risk for SGA in women with SVT (aRR = 5.93, 95%CI [1.23, 28.55]), with no significant associations reported for AF/AFl or PVCs after adjustment (Supplementary Figure S14).

#### 3.4.9. Neonatal Death

Three studies provided raw data and were included in this analysis [14,23,27]. The overall risk of neonatal death was not significantly increased in pregnancies complicated by any type of arrhythmia (RR = 3.76, 95%CI [0.35, 40.64]), an analysis with substantial heterogeneity (I^2^ = 74%). However, the subgroup analysis revealed that maternal VT/VF was associated with a significant 15-fold increased risk of neonatal death (RR = 15.47, 95%CI [3.45, 69.45]). AF/AFl was not associated with a significant increase in the risk for neonatal mortality. The test for subgroup differences confirmed that the risk of neonatal death varied significantly among arrhythmia subtypes (*p* = 0.04) (Supplementary Figure S15).

#### 3.4.10. Five-Minute Apgar Score

Five studies provided raw data and were included in this analysis [14,20,21,23,27]. Maternal cardiac arrhythmias were not associated with a 5 min Apgar score < 7 (RR = 1.66, 95%CI [0.66, 4.16]) and were characterized by substantial heterogeneity (I^2^ = 67%). However, subgroup analysis showed that VT/VF was associated with a significant 3.6-fold increased risk of a low Apgar score (RR = 3.60, 95%CI [2.13, 6.07]). No significant associations were found for the mixed arrhythmias cohort, AF/AFl, or PVCs. The test for subgroup differences confirmed that the risk varied significantly among arrhythmia subtypes (*p* = 0.01) (Supplementary Figure S16).

#### 3.4.11. Neonatal Intensive Care Unit Admission

Six studies provided raw data and were included in this analysis [13,21,23,24,28,31]. The overall risk of NICU admission was not significantly increased in pregnancies complicated with any maternal arrhythmia, showing a borderline effect (RR = 2.10, 95%CI [0.99, 4.48]), with substantial heterogeneity across studies (I^2^ = 76%). In the subgroup analyses, no statistically significant increase in risk was observed among the different arrhythmia subtypes (*p* = 0.77) (Supplementary Figure S17). In the sensitivity analysis for NICU admission using adjusted estimates, the overall risk was significantly increased (aRR = 26.74, 95%CI [2.36, 303.41]). The adjusted analysis revealed a significant increase in NICU risk among women with SVT (aRR = 49.60, 95%CI [5.08, 484.28]) and VT/VF (aRR = 124.80, 95%CI [14.26, 1092.24]). No significant associations were found for AF/AFl after adjustment (Supplementary Figure S18).

The cumulative results of the above analyses are presented in Table 3 and Table 4.

### 3.5. Publication Bias

**Figure 6:**
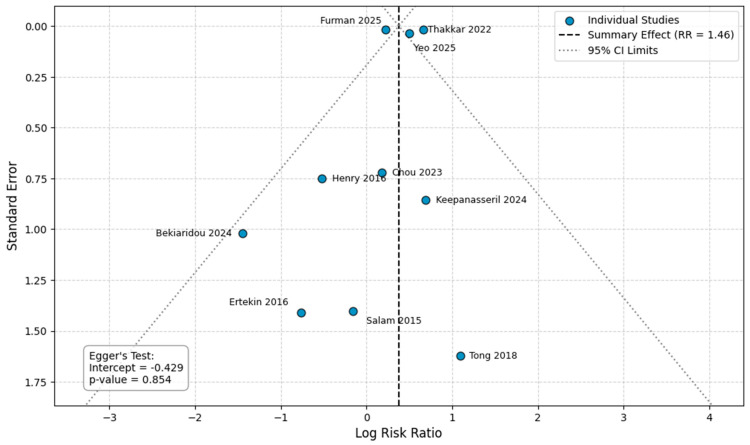
Publication bias analysis for preeclampsia. Abbreviations: CI, confidence intervals; RR, Risk Ratio [12,13,14,15,20,21,23,27,29,30].

**Figure 7:**
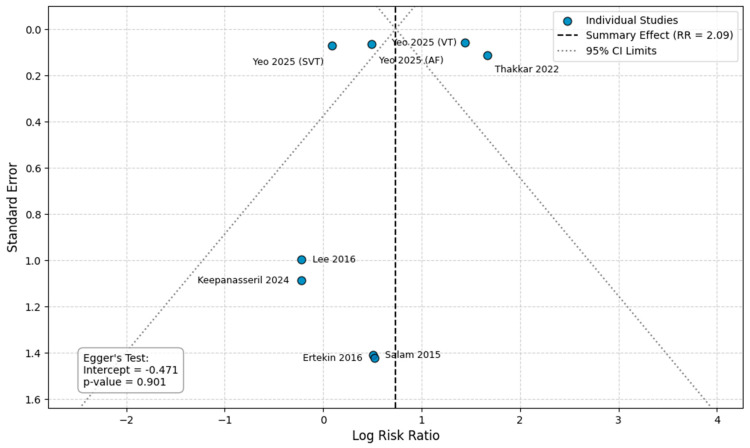
Publication bias analysis for stillbirth. Abbreviations: CI, confidence intervals; RR, Risk Ratio [14,15,23,24,27,29].

**Figure 8:**
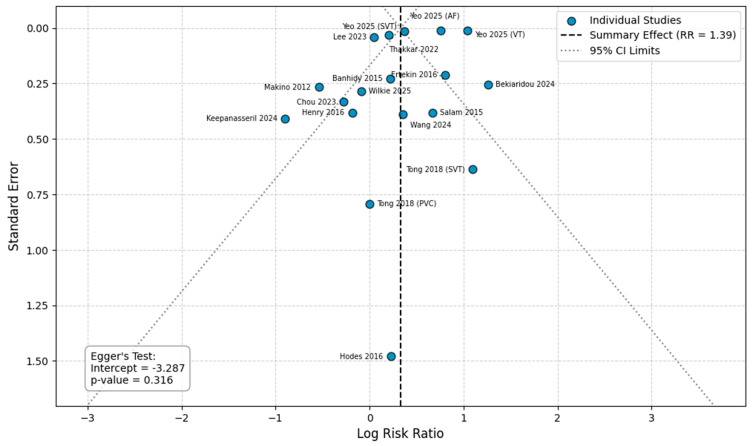
Publication bias analysis for preterm delivery. Abbreviations: CI, confidence intervals; RR, Risk Ratio [13,14,15,18,20,21,22,23,25,26,27,29,30,31,32].

**Figure 9:**
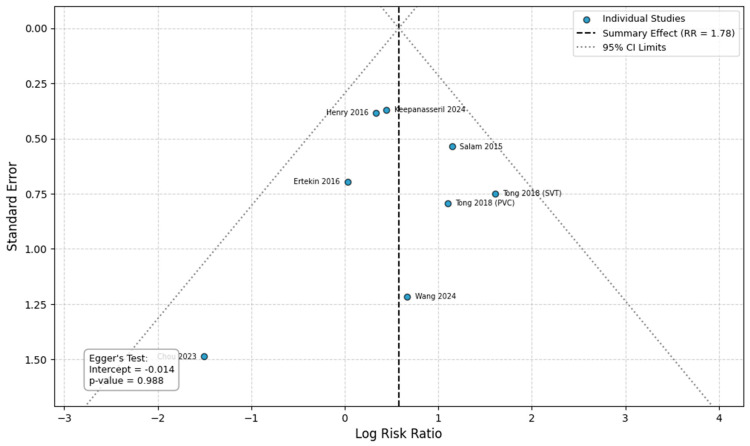
Publication bias analysis for SGA. Abbreviations: CI, confidence intervals; RR, Risk Ratio [14,20,21,23,27,30,31].

Visual inspection of the funnel plots for the primary outcomes did not reveal any distinct asymmetry (Figure 6, Figure 7, Figure 8 and Figure 9). Egger’s regression test was performed for the outcomes with a sufficient number of included studies (*n* ≥ 10) and confirmed the absence of significant small-study effects. Specifically, the analysis for preeclampsia (10 studies, intercept = −0.429, *p* = 0.854) and preterm delivery (15 studies; intercept = −3.287, *p* = 0.316) yielded a non-significant intercept, indicating no evident publication bias. For stillbirth and SGA, the analyses included fewer than 10 studies (8 comparisons each) and therefore the Egger’s test was considered underpowered to reliably detect asymmetry (stillbirth: intercept = −0.471, *p* = 0.90; SGA: intercept = −0.014, *p* = 0.988).

## 4. Discussion

### 4.1. Summary of Principal Findings

This study established several key findings; first, the presence of any maternal cardiac arrhythmia was significantly associated with an increased overall risk for adverse pregnancy outcomes, including preeclampsia, placental abruption, cesarean section, preterm delivery, and SGA neonates. Second, it revealed a clear pattern of risk stratification among arrhythmia subtypes; maternal VT/VF emerged as the most dangerous subtype for the fetus and neonate, associated with the highest risk for the most severe outcomes, including a more than four-fold increase in stillbirth and a fifteen-fold increase in neonatal death. Third, SVT and AF/AFl were also linked to significant perinatal consequences; SVT was most strongly associated with SGA neonates and showed a significant link to preterm delivery, while AF/AFl was also a significant predictor of SGA neonates and stillbirth. Finally, our sensitivity analyses, which pooled adjusted effect estimates, consistently reinforced these associations, highlighting the robustness of the findings after accounting for potential confounding variables.

### 4.2. Interpretation of Results

Our results support the hypothesis that a tachyarrhythmia may compromise the effective cardiac output [33,34], which in turn affects placental blood flow and is associated with fetal hypoxia and a spectrum of adverse outcomes. Recent meta-analytic data demonstrated that maternal cardiac output begins to rise in the first trimester, driven largely by a reduction in systemic vascular resistance and a corresponding increase in heart rate, and continues to increase until it peaks in the early third trimester at approximately 30% above non-pregnant values [35]. The consequences of this disruption manifested across a spectrum of outcomes suggestive of placental insufficiency; the most severe of these, stillbirth, was significantly doubled overall. The profound risk conferred by VT/VF (RR = 4.20) likely reflects acute, catastrophic reductions in uteroplacental perfusion during episodes of severe hemodynamic compromise [33]. However, these events almost invariably occur in the context of significant structural heart disease, suggesting that the underlying myocardial pathology is the primary driver of mortality [14]. The significant risk from AF/AFl (RR = 1.62) may stem from a dual mechanism: hemodynamic instability due to the loss of atrial systolic function and a heightened risk of thromboembolic events within the placental vasculature, amplified by the prothrombotic pregnancy status [27,36,37,38]. However, it is imperative to interpret these findings in the context of the underlying substrate. AF in pregnancy is frequently a consequence of structural heart pathology, particularly rheumatic mitral valve disease [23,27]. Therefore, the adverse outcomes observed in the AF/AFl group may largely reflect the severity of the underlying valvular disease rather than the rhythm disturbance itself.

A pattern of chronic placental insufficiency among women with cardiac arrhythmias may be indicative of the increased risk related to preterm delivery and SGA neonates. The significant risk of preterm delivery associated with maternal VT/VF (RR = 2.84) likely resembles the pathophysiologic mechanism that has been proposed for stillbirth; episodes of severe hemodynamic compromise, while not fatal, can induce sufficient placental stress to trigger premature labor [33]. The strong association of maternal SVT with both SGA neonates (RR = 3.86) and preterm delivery (RR = 1.78) probably highlights a different pathway. SVT episodes may manifest with subtle symptoms that may be perceived as normal pregnancy complaints and therefore persist for a longer period till cardioversion to sinus rhythm [39]. These sustained or recurrent episodes, even if not critically compromising hemodynamics at any single moment, can create a state of chronic, cumulative oxygen and nutrient deficit that impairs fetal growth [34]. However, to interpret this association requires distinguishing the consequences of disease itself from its medical treatment. The management of SVT itself introduces a potential confounder. Beta-blocker therapy, commonly used for prevention of recurrent episodes of SVT, has been associated with fetal growth restriction [40,41]. While our adjusted analysis suggests the arrhythmia plays a role, current data do not allow us to fully disentangle the medication effect from the hemodynamic effect of the arrhythmia due to a lack of dosage and adherence data in primary studies. Thus, the observed association with SGA may represent a composite of hemodynamic impairment and pharmacological effects. The rapid ventricular response is linked to hemodynamic instability by shortening diastolic filling time, which reduces overall cardiac output [42]. This chronic reduction in cardiac efficiency, paired with the risk of placental microthrombosis from atrial stasis, can progressively impair fetal growth over time [23].

Beyond these direct hemodynamic consequences, the relationship between maternal arrhythmias and preeclampsia suggests a shared underlying vascular pathology. While the mixed arrhythmias cohort showed a significant association with preeclampsia (RR = 1.77) in the context of placental insufficiency, no specific arrhythmia had a significant association on its own in the unadjusted analysis. However, the observation that the overall risk for preeclampsia remained statistically significant after adjustment (aRR = 1.14), a finding predominantly reflected in the large SVT cohort (aRR = 1.14), points to a mechanism deeper than simple confounding by shared risk factors. SVT may act as a clinical marker, potentially associated with pre-existing, subclinical cardiovascular dysfunction, such as endothelial stress or vascular stiffness, which is unmasked when faced with the profound hemodynamic load of pregnancy [12,13]. This specific link between SVT and preeclampsia, which persisted after adjustment while general hypertensive disorders did not, is best explained by shared autonomic dysfunction [43]. Thus, we hypothesize that SVT in a structurally normal heart might not merely be a comorbidity, but rather a potential early clinical manifestation of the same vascular maladaptation that is associated with placental ischemia. This concept of shared pathophysiology is further reinforced by long-term data. Studies show that preeclampsia itself acts as a female-specific cardiovascular risk enhancer, significantly increasing the long-term risk of developing arrhythmias later in life [44]. This longitudinal association provides further proof that both the arrhythmia observed during pregnancy and the vascular disease of preeclampsia may be clinical manifestations of the same underlying cardiovascular vulnerability [44]. Both preeclampsia and cardiac arrhythmias share a common root in vascular inflammation, oxidative stress, and endothelial dysfunction [44]. This concept of underlying vulnerability is illustrated by our subgroup analysis. We observed a paradox where the risk of preeclampsia was significantly elevated in the general population cohort (RR = 1.59) but not in women with known cardiac disease. This discrepancy supports the ‘unmasking’ hypothesis. Women with known cardiac disease enter pregnancy under strict surveillance and optimized medical therapy. In contrast, women in the general population who develop arrhythmias likely have occult cardiovascular disease that is only revealed by the stress of pregnancy, presenting simultaneously as arrhythmic events and preeclampsia without the benefit of prior prophylaxis [44]. Furthermore, it is likely that preconception factors, such as obesity, advanced maternal age, and substance use, contribute to this ‘unmasking’ effect by creating a vulnerable cardiovascular substrate that manifests only under the physiological stress of pregnancy [5].

Although neither overall neonatal mortality nor low 5 min Apgar scores reached statistical significance across the full cohort, both outcomes showed sharply elevated risk among women with VT/VF. However, it is critical to contextualize these findings, as the data for this specific subgroup were derived from a single study exclusively involving women with structural heart disease [14], limiting generalizability to the broader obstetric population. While the relative risk is high (RR > 15), the absolute event rate of neonatal death in this cohort was 4.8% (2/42) compared to 0.3% (9/2924) in the control group [14]. These findings should therefore be interpreted as identifying a high-risk subpopulation requiring specialized care, rather than implying a common occurrence in the general obstetric population. VT/VF can precipitate acute hemodynamic collapse, sharply reducing uteroplacental perfusion, a state highly correlated with fetal hypoxia. This acute insult may set the stage for neonatal compromise after birth. Second, low 5 min Apgar score and neonatal mortality may reflect the iatrogenic consequences of prematurity. The same severe physiologic stress that triggers fetal risk often prompts clinicians to pursue preterm delivery, both to limit ongoing hemodynamic instability due to maternal VT/VF and to allow administration of antiarrhythmic drugs that may be unsafe for the fetus (e.g., amiodarone). These necessary interventions, however, expose the neonate to the inherent risks of premature birth, amplifying the mortality risk triggered by the maternal arrhythmia [14].

The analysis of NICU admissions highlights the importance of accounting for potential confounders. Although the unadjusted overall risk for NICU admission was not significant, the adjusted risk was elevated (aRR = 26.74) after controlling for confounding factors. This elevated risk was driven primarily by the VT/VF (aRR = 124.80) and the SVT subgroups (aRR = 49.60). The extremely high risk observed in the VT/VF suggests that affected neonates often experience acute peripartum cardiorespiratory compromise, necessitating immediate intensive care [14]. In case of a repeated sustained VT early cesarean delivery is usually opted to improve maternal and fetal survival [7,9]. In contrast, the increased risk of NICU associated with SVT appears to stem from chronic fetal stress, contributing to SGA neonates and preterm delivery [13]. These conditions predispose infants to respiratory, infectious, and metabolic complications that commonly require NICU support. Overall, NICU admission serves as a meaningful indicator of the substantial, non-lethal neonatal morbidity linked to maternal tachyarrhythmias.

The analysis of systemic hypertensive and metabolic complications suggests the primary influence of shared underlying maternal risk factors. The significant overall unadjusted risk for hypertensive disorders of pregnancy (RR = 1.25) disappeared after adjustment (aRR = 1.03). This result strongly suggests that the crude association is driven by maternal comorbidities, such as advanced maternal age and obesity, that predispose women to both arrhythmias and hypertension. Similarly, the overall risk for GDM was not significant (RR = 1.42), aligning with the conclusion that the link is largely due to shared cardiovascular comorbidities [1,45].

Correspondingly, placental abruption demonstrated a substantially elevated risk in women with any type of arrhythmia (RR = 2.59), but not among specific arrhythmia subtypes. Placental abruption often represents the terminal and most severe manifestation of target-organ vascular dysfunction, and gestational hypertension is among its most common and potent antecedents [46]. Thus, the observed association suggests that tachyarrhythmias may serve as markers of a heavier cardiovascular substrate, such as metabolic syndrome. This comorbidity likely constitutes the true pathophysiologic driver of both the development of gestational hypertension and, subsequently, the increased risk of placental abruption.

A significant increase in the risk of CS was found across all pregnancies complicated by maternal arrhythmia (RR = 1.27). Women with VT/VF or SVT demonstrated a higher risk of delivering with CS. Furthermore, SVT remained a significant risk factor even after adjustment for major confounders, suggesting that SVT may confer an inherent obstetric vulnerability that cannot be explained solely by underlying maternal comorbidities (such as advanced age or chronic medical conditions) [13]. This association is further supported by the observation that the risk for CS was elevated both in the general population of pregnant women (RR = 1.36) and among women with pre-existing cardiac disease (RR = 1.31). Thus, the presence of the arrhythmia itself, rather than the severity of the underlying cardiac substrate, appears to be the primary factor influencing clinical decision-making. The sustained and/or recurrent nature of SVT likely imposes sufficient hemodynamic stress, or generates enough clinical concern during labor, to prompt clinicians to proceed with CS to prevent maternal cardiac decompensation or fetal compromise. Finally, in the context of delivery management, data reveal a vital distinction between sustained tachyarrhythmias and ectopic beats. PVCs were associated with a significantly decreased risk of cesarean section by 25%, a protective effect that strengthened after adjustment. It is plausible that this phenomenon is behavioral rather than biological. Unlike the urgency associated with VT/VF or SVT, the detection of PVCs typically leads to reassurance [47]. This de-escalation prevents the ‘cascade of intervention’ and anxiety-driven operative deliveries that characterize the management of complex arrhythmias, suggesting that accurate arrhythmia subtyping is as critical for preventing unnecessary interventions as it is for treating pathology.

### 4.3. Clinical Significance of Findings

The results of this meta-analysis suggest potential implications for the clinical management of pregnant women, extending the focus of arrhythmia management beyond maternal stability to consider fetal well-being. First, the association of SVT with preeclampsia and SGA neonates indicates that SVT is not as benign as it was considered to be, both for the mother and the fetus. Consequently, closer observation of maternal blood pressure and fetal growth may be offered to these women. Future research should investigate whether enhanced surveillance protocols could improve outcomes. Second, risk stratification for perinatal morbidity highlights the importance of appropriate care settings. The increase in NICU admissions observed in the adjusted analyses for both VT/VF and SVT subgroups, alongside the elevated risk of neonatal death associated with VT/VF suggests that these arrhythmias may carry significant fetal risks. Therefore, while our observational data cannot dictate clinical pathways, it raises the question of whether managing pregnant women with SVT at centers equipped with neonatal intensive care units might be beneficial, a concept that warrants future prospective evaluation. Finally, the “unmasking” phenomenon observed in our subgroup analysis, where women in the general population often faced higher risk for adverse pregnancy outcomes than those with known heart disease, raises questions about current risk stratification. The onset of a new arrhythmia in a previously low-risk pregnant woman may potentially signal occult cardiovascular or placental vulnerability. Therefore, a multidisciplinary evaluation may be beneficial to rule out underlying pathology. However, further studies are needed to confirm, if such an approach alters clinical outcomes.

### 4.4. Strengths and Limitations

This meta-analysis has several notable strengths. To our knowledge, it represents the most comprehensive quantitative synthesis to date examining the association between maternal arrhythmias and obstetric and perinatal outcomes. Through detailed subgroup analyses, we delineated distinct associations for SVT, AF/AFl, VT/VF, and PVCs, thereby providing clinically meaningful insights rather than treating arrhythmias as a homogeneous entity.

Furthermore, studies assessed as having a higher risk of bias—primarily due to inadequate adjustment for key confounders—likely overestimated the crude risk of adverse outcomes. By prioritizing adjusted estimates, our analysis offers a more conservative and robust assessment of the true associations.

However, our findings should be interpreted in light of several important limitations. First, the majority of included studies were observational and retrospective in design, introducing inherent risks of selection bias, residual confounding, and reliance on administrative coding accuracy, which may result in misclassification of arrhythmia diagnoses.

Second, substantial statistical heterogeneity was observed across several outcomes. Although subgroup analyses indicated that much of this variability was attributable to the specific arrhythmia type, additional factors—such as differences in baseline cardiovascular risk profiles, diagnostic approaches, and obstetric management protocols across institutions—likely contributed. Notably, the limited number of studies within each subgroup, combined with a lack of granular covariate data, precluded the use of meta-regression to further investigate these sources of heterogeneity.

Third, with respect to the underlying cardiac substrate, we stratified outcomes by the presence of known heart disease versus the general population. However, inconsistent reporting of specific structural lesions (e.g., rheumatic mitral valve disease) across primary studies prevented quantitative subgroup analyses. While available data on structural conditions, arrhythmia duration, and adequacy of rate control are summarized qualitatively in Supplementary Table S1, the lack of standardized, detailed data limited our ability to disentangle the independent effects of arrhythmia from the severity of underlying structural or valvular pathology.

Fourth, similar inconsistencies precluded quantitative adjustment for antiarrhythmic drug dosing, medication adherence, and precise arrhythmia burden. Consequently, the potential contribution of therapies—such as beta-blockers—to outcomes like fetal growth restriction remains a source of residual confounding.

Fifth, preconception lifestyle factors, including tobacco and alcohol use, were not consistently reported, preventing adjustment for these variables despite their established impact on pregnancy outcomes.

Finally, outcome definitions (e.g., small for gestational age and preterm delivery) varied across studies, and limited temporal resolution in many primary sources precluded analysis of whether arrhythmias occurred proximal to, or remote from, adverse outcomes. Retrospective harmonization of these heterogeneous definitions was not feasible.

## 5. Conclusions

This meta-analysis demonstrated that maternal cardiac arrhythmias were associated with adverse pregnancy outcomes. We identified two key patterns. VT/VF was linked to an increased risk of stillbirth and neonatal death, potentially reflecting acute hemodynamic compromise, whereas SVT showed a robust association with preeclampsia and SGA neonates, which may signal underlying chronic vascular dysfunction or shared risk factors. The observation that these risks were often higher in the general population supports the concept of pregnancy potentially unmasking occult cardiovascular disease. Consequently, the presence of an arrhythmia may serve as a clinical marker for placental insufficiency, suggesting that such pregnancies might benefit from closer surveillance.

## Figures and Tables

**Table 1:** Characteristics of the included studies.

Study	Period	Type	Country	Inclusion Criteria	Exclusion Criteria	Case Group	Control Group	Arrhythmias (Diagnosis)	Outcomes
Banhidy et al., 2015 [18]	1980–1996	Retrospective Cohort	Hungary	Pregnant women in Hungarian Case–Control Surveillance of Congenital Abnormalities database Cases had medically recorded paroxysmal SVT Live-born offspring without congenital abnormalities	Fetal congenital abnormalities Mothers with other arrhythmias or structural heart disease	Pregnant women with paroxysmal SVT (*n* = 149) Age = 26.4 ± 5.2 y	Pregnant women without paroxysmal SVT (general pop.) (*n* = 38,002) Age = 25.5 ± 4.9 y	Paroxysmal SVT (Medical records)	GA at delivery Birthweight PTD
Bekiaridou et al., 2024 [13]	2015–2022	Retrospective Cohort	USA	No known structural heart disease Primiparous/multiparous without prior CS	History of: CMP, coronary artery disease, ischemic heart disease, myocarditis, VHD, CHD, RHD, or cardiac device	Pregnant women with documented SVT (*n* = 76) Age = 33.2 ± 4.8 y	Healthy pregnant women without SVT (4:1 ratio) (*n* = 304) Age = 32.1 ± 4.4 y	SVT (Electrocardiographic confirmation)	CS PTD NICU admission
Chang et al., 2017 [19]	2001–2012	Retrospective Cohort	Taiwan	All pregnancies in a national insurance database	Age < 15 or >44 years CHD Delivery gap < 6 mo or >20 years Multiparity	Pregnant women with symptomatic paroxysmal SVT (emergency department visit/admission) (*n* = 769) Mean age = 30 y	Pregnant women without paroxysmal SVT (general pop.) (*n* = 2,349,559) Mean age = 30 y	Paroxysmal SVT (Administrative coding)	Obstetric: CS, hypertensive disorders, GDM Perinatal: PTD, stillbirth
Chou et al., 2023 [20]	2005–2020	Retrospective Cohort	Taiwan	Singleton pregnancies with delivery/termination and ≥1 prenatal visit	Age < 18 years Incomplete records Structural heart disease, sick sinus syndrome, atrioventricular block, HF, other tachyarrhythmias Prior cardiac surgery/ablation/device	Pregnant women with PVC burden ≥ 1% on Holter (*n* = 107) Age = 34.2 ± 4.3 y	Healthy pregnant women without PVCs (or other major diseases) (*n* = 214) Age = 34.0 ± 3.6 y	PVCs (Electrocardiographic confirmation)	Obstetric: Placental abruption, preeclampsia, gestational hypertension, GDM Perinatal: Apgar < 7, PTD, SGA, stillbirth
Ertekin et al., 2016 [14]	2007–2013	Retrospective Cohort	Multinational	Pregnant women with CHD, VHD, CMP, ischemic heart disease, aortic pathology, or pulmonary hypertension	Arrhythmia in a structurally normal heart	Pregnant women with structural heart disease who developed VT (*n* = 42) Age = 28.9 ± 5.7 y	Pregnant women with structural heart disease who did not develop VT (*n* = 2924) Age = 29.3 ± 5.6 y	VA (Clinical records)	Obstetric: PIH, preeclampsia, CS Perinatal: SGA, fetal/neonatal death, Apgar < 7, PTD
Furman et al., 2025 [12]	2016–2021	Retrospective Cohort	USA	Pregnancy-related hospitalizations with a diagnosis of SVT	Not specified	Pregnant women hospitalized with SVT (*n* = 30,215) Age = 29.81 (29.65–29.97) y	Pregnant women hospitalized without SVT (*n* = 23,500,000) Age = 29.01 (28.96–29.05) y	SVT (Administrative coding)	Preeclampsia
Henry et al., 2016 [21]	2008–2013	Retrospective Cohort	USA	Pregnant women with maternal cardiac disease in Pregnancy and Cardiac Treatment program	Multiple pregnancies (only first included)	Women with maternal cardiac disease and arrhythmia (*n* = 36) Age = 31.5 ± 5.6	Women with maternal cardiac disease and no arrhythmia (*n* = 107) Age = 30.2 ± 6.7)	SVT, AF, VT, WPW, Arrhythmogenic right ventricular dysplasia (Electrocardiographic confirmation)	Obstetric: CS, intrauterine fetal demise, preeclampsia, GDM, placental abruption Perinatal: PTD, NICU admission, Apgar < 7, SGA
Hodes et al., 2016 [22]	N/A	Prospective Cohort	Netherlands/USA	Pregnant patients (15–45 years) with arrhythmogenic right ventricular dysplasia/CMP meeting 2010 Task Force Criteria	First-trimester fetal loss	Pregnant women with arrhythmogenic right ventricular dysplasia/CMP who experienced sustained VA (*n* = 5) Age = 31.7 ± 1.9	Pregnant women with arrhythmogenic right ventricular dysplasia/CMP who had HF or no major cardiac event HF group;(*n* = 2) Age = 34.4 ± 4.7 No major event group; (*n* = 32) Age = 31.0 ± 3.7)	Sustained VA (Electrocardiographic/Device confirmation)	Stillbirth PTD
Keepanasseril et al., 2024 [23]	2011–2015	Retrospective Cohort	India	Pregnant women with RHD and AF	Not specified	Pregnant women with RHD and AF (*n* = 71) Age = 27.3 ± 4.5 y	Pregnant women with RHD without AF (*n* = 284) Age = 27.1 ± 4.7 y	AF (Electrocardiographic confirmation)	Obstetric: Hypertensive disorders of pregnancy, GDM Perinatal: stillbirth, SGA, PTD, CS, NICU admission, neonatal death
Lee et al., 2016 [24]	2003–2013	Retrospective Cohort	USA	All births in Kaiser Permanente Southern California Health System	Not continuously enrolled in health plan	Pregnant women with AF/AFl (*n* = 157) Age = 32.8 ± 5.2	Pregnant women without AF/AFl (general pop.) (*n* = 264,573) Age = 29.8 ± 5.9	AF, AFl (Administrative coding)	Obstetric: PIH, preeclampsia, abruption Perinatal: PTD, premature rupture of membranes, SGA, demise, neonatal death, NICU admission
Lee et al., 2023 [25]	2017	Retrospective Cohort	S. Korea	Women aged 25–40 who delivered in 2017	Not specified	Women with any of the investigated arrhythmias (*n* = 7312) Age NR	Women without arrhythmia (*n* = 154,913) Age NR	Conduction disorder, WPW, SVT, AF/AFl, VA, sick sinus syndrome (Administrative coding)	PTD
Makino et al., 2012 [26]	1991–2005	Retrospective Case–Control	Japan	Pregnant women with cardiac disease (congenital, acquired, arrhythmia, CMP)	Not specified	Women with any of the investigated arrhythmias (*n* = 186) Age NR	Women without arrhythmia (*n* = 413) Age NR	PVCs, atrioventricular block, WPW, paroxysmal SVT, long QT syndrome, premature atrial contraction, sick sinus syndrome, VT (Medical records/Electrocardiographic confirmation)	PTD
Salam et al., 2015 [27]	2007–2011	Prospective & Retrospective Cohort	Multinational	Pregnant patients with structural heart disease (from ROPAC registry)	Non-structural heart disease	Women with structural heart disease who developed AF/AFl pregnancy (*n* = 17) Age = 32 ± 5.0 y	Women with structural heart disease who did not develop AF/AFl (*n* = 1304) Age = 30 ± 5.6 y	AF, AFl (Clinical records)	Obstetric: PIH, preeclampsia, CS Perinatal: Stillbirth, SGA, Apgar < 7, PTD
Sharma et al., 2022 [28]	2015–2021	Retrospective Cohort	USA	Women admitted for delivery with no known structural heart disease	Structural heart disease Multiple deliveries (only first included) Arrhythmia during cardiopulmonary resuscitation	Women with structurally normal hearts with a perilabour arrhythmic event (*n* = 137) Age = 31.72 ± 4.93	Women with structurally normal hearts without arrhythmia(*n* = 1031) Age = 31.37 ± 5.42	SVT, AF/AFl, VA (non-sustained VT or frequent PVCs) (Electrocardiographic confirmation)	CS PTD NICU admission
Thakkar et al., 2022 [29]	2009–2019	Retrospective Cohort	USA	Pregnant patients ≥ 18 years hospitalized for delivery	Not specified	Hospitalized pregnant women with arrhythmia (*n* = 36,442) Age NR	Hospitalized pregnant women without arrhythmia (general pop.) (*n* = 41,540,000) Age NR	Arrhythmias in general, including SVT, AF, AFl, VT, VF (Administrative coding)	Stillbirth CS
Tong et al., 2018 [30]	2010–2016	Prospective Case–Control	Canada	Pregnant patients referred for PVCs	PVC burden < 1% Spontaneous abortion < 20 wks Structural heart disease History of cardiovascular events	(1) Pts with PVC > 1% (2) Pts with SVT (PVC < 1%) (PVC cases = 53, SVT cases = 53) Age NR	Low-risk normal pregnancy with no cardiac diagnosis (*n* = 53) Age NR	PVCs, SVT as different study groups (Electrocardiographic confirmation)	Obstetric: PIH Perinatal: PTD, SGA, fetal/neonatal death
Wang et al., 2024 [31]	2011–2021	Retrospective Cohort	China	Pregnant women hospitalized with pre-excitation syndrome	Voluntary pregnancy termination	Pregnant women with pre-excitation syndrome who developed SVT (*n* = 65) Age = 31.1 ± 4.4	Pregnant women with pre-excitation syndrome who did not develop SVT (*n* = 254) Age = 30.6 ± 4.6	SVT (Electrocardiographic confirmation)	Obstetric: GDM, preeclampsia Perinatal: PTD, SGA, NICU transfer, stillbirth
Wilkie et al., 2025 [32]	2021–2025	Retrospective Cohort	USA	Pregnant individuals with Ebstein’s anomaly	Other CHD	Pregnant patients with Ebstein’s anomaly who developed an arrhythmia (*n* = 108) Age = 32.0 ± 5 y	Pregnant patients with Ebstein’s anomaly without arrhythmia (*n* = 153) Age = 32.0 ± 6 y	AF/AFl, SVT, VA, PVC, bradyarrhythmias (Administrative coding/Clinical records)	PTD, CS
Yeo et al., 2025 [15]	2017–2020	Retrospective Cohort	USA	Women ≥ 18 years admitted for delivery	Missing data on GA or delivery outcomes	Patients with cardiac tachyarrhythmias (SVT, AF/AFl, VA) (*n* = 11,091) Age = 30.5 ± 5.8	Patients without cardiac tachyarrhythmias (general pop.) (*n*= 7,506,884) Age = 29.3 ± 5.7	SVT, AF/AFl, VA (Administrative coding)	Stillbirth, PTD

Abbreviations: AF, atrial fibrillation; AFl, atrial flutter; CHD, congenital heart disease; CMP, cardiomyopathy; CS, Cesarean section; GA, gestational age; GDM, gestational diabetes mellitus; HF, heart failure; mo, months; NICU, neonatal intensive care unit; NR, not reported; PIH, pregnancy-induced hypertension; pop., population; PTD, preterm delivery; PVC, premature ventricular contractions; RHD, rheumatic heart disease; SGA, small for gestational age; SVT, supraventricular tachycardia; VA, ventricular arrhythmia; VHD, valvular heart disease; VF, ventricular fibrillation; VT, ventricular tachycardia; wks, weeks; WPW, Wolff–Parkinson–White syndrome; y, years.

**Table 2:** Quality of the included studies according to Newcastle–Ottawa Scale.

First Author, Year	Study Type	S1	S2	S3	S4	C	O1	O2	O3	Total
Banhidy et al., 2015 [18]	Retrospective cohort	b *	a *	a *	a *	a,b **	b *	a *	a *	9
Bekiaridou et al., 2024 [13]	Retrospective cohort	a *	a *	a *	a *	a *	a *	a *	a *	8
Chang et al., 2017 [19]	Retrospective cohort	a *	a *	a *	a *	a,b **	b *	a *	a *	9
Chou et al., 2023 [20]	Retrospective cohort	b *	a *	a *	a *	a,b **	b *	a *	a *	9
Ertekin et al., 2016 [14]	Retrospective cohort	b *	a *	a *	a *	a *	b *	a *	a *	8
Furman et al., 2025 [12]	Retrospective cohort	a *	a *	a *	a *	a,b **	a *	a *	a *	9
Henry et al., 2016 [21]	Retrospective cohort	b *	a *	a *	a *	a,b **	b *	a *	a *	9
Hodes et al., 2016 [22]	prospective cohort	b *	a *	a *	a *	-	b *	a *	a *	7
Keepanasseril et al., 2024 [23]	Retrospective cohort	b *	a *	a *	a *	a,b **	a *	a *	a *	9
Lee et al., 2016 [24]	Retrospective cohort	b *	a *	a *	a *	-	b *	a *	a *	7
Lee et al., 2023 [25]	Retrospective cohort	a *	a *	a *	a *	-	a *	a *	a *	7
Makino et al., 2012 [26]	Retrospective cohort	b *	a *	a *	a *	-	b *	a *	a *	7
Salam et al., 2015 [27]	Retrospective cohort	b *	a *	a *	a *	-	b *	a *	a *	7
Sharma et al., 2022 [28]	Retrospective case–control	a *	a *	b	a *	a,b **	a *	a *	a *	8
Thakkar et al., 2022 [29]	Retrospective cohort	a *	a *	a *	a *	a,b **	b *	a *	a *	9
Tong et al., 2018 [30]	Prospective case–control study	a *	a *	b	a *	a *	a *	a *	a *	7
Wang et al., 2024 [31]	Retrospective cohort	b *	a *	a *	a *	a *	b *	a *	a *	8
Wilkie et al., 2025 [32]	Retrospective cohort	b *	a *	a *	a *	a *	b *	a *	a *	8
Yeo et al., 2025 [15]	Retrospective cohort	a *	a *	a *	a *	a,b **	a *	a *	a *	9

Abbreviations: a, first answer according to Newcastle–Ottawa Scale (NOS); b, second answer according to NOS; S, selection; C, comparability; O, outcome; *, attribution of a star according to NOS; **, attribution of two stars according to NOS.

**Table 3:** Summary of pooled risk ratios for primary arrhythmia subgroup analyses.

Outcome	Overall (RR [95% CI])	Mixed Arrhythmias Cohort	AF/AFl	SVT	VT/VF	PVCs
Preeclampsia	1.46 [1.10, 1.93]	1.77 [1.50, 2.09]	1.58 [0.38, 6.60]	0.74 [0.16, 3.38]	0.46 [0.03, 7.35]	1.40 [0.38, 5.07]
Hypertensive Disorders	1.25 [1.17, 1.33]	1.25 [1.17, 1.33]	0.80 [0.22, 2.94]	6.00 [0.75, 48.15]	0.48 [0.03, 7.66]	1.06 [0.27, 4.15]
Gestational Diabetes	1.42 [0.94, 2.16]	1.45 [0.83, 2.54]	0.74 [0.34, 1.58]	1.81 [1.16, 2.84]	-	2.00 [0.66, 6.05]
Cesarean Section	1.27 [1.13, 1.42]	1.35 [1.20, 1.51]	1.15 [0.80, 1.65]	1.38 [1.23, 1.56]	1.47 [1.20, 1.81]	0.75 [0.59, 0.97]
Placental Abruption	2.59 [2.29, 2.93]	3.11 [1.08, 8.97]	-	2.66 [0.45, 15.55]	-	9.95 [0.48, 205.51]
Stillbirth	2.09 [1.11, 3.91]	5.28 [4.22, 6.61]	1.62 [1.42, 1.84]	1.09 [0.95, 1.26]	4.20 [3.75, 4.71]	-
Preterm delivery	1.39 [1.12, 1.72]	1.05 [0.89, 1.23]	1.26 [0.50, 3.16]	1.78 [1.21, 2.62]	2.84 [2.77, 2.90]	0.79 [0.43, 1.45]
SGA	1.78 [1.20, 2.66]	1.40 [0.66, 2.96]	2.00 [1.03, 3.91]	3.86 [1.10, 13.49]	1.03 [0.26, 4.03]	1.09 [0.08, 13.92]
Neonatal death	3.76 [0.35, 40.64]	-	1.35 [0.21, 8.55]	-	15.47 [3.45, 69.45]	-
5 min Apgar < 7	1.66 [0.66, 4.16]	1.49 [0.60, 3.67]	0.64 [0.23, 1.77]	-	3.60 [2.13, 6.07]	9.95 [0.48, 205.51]
NICU admission	2.10 [0.99, 4.48]	7.45 [0.08, 733.66]	1.59 [0.72, 3.49]	1.99 [0.88, 4.49]	-	-

Abbreviations: AF, atrial fibrillation; AFl, atrial flutter; CI, Confidence Interval; NICU, neonatal intensive care unit; PVCs, premature ventricular contractions; RR, Risk Ratio; SGA, small for gestational age; SVT, supraventricular tachycardia; VF, ventricular fibrillation; VT, ventricular tachycardia.

**Table 4:** Summary of pooled adjusted risk ratios for arrhythmia subgroup analyses.

Outcome	Overall (aRR [95% CI])	AF/AFl	SVT	VT/VF	PVCs
Preeclampsia	1.14 [1.04, 1.25]	2.03 [0.36, 11.30]	1.14 [1.04, 1.24]	-	1.41 [0.38, 5.29]
Hypertensive Disorders	1.03 [0.59, 1.78]	0.72 [0.16, 3.32]	1.85 [0.29, 11.79]	-	1.07 [0.26, 4.39]
Gestational Diabetes	1.05 [0.76, 1.44]	0.71 [0.30, 1.66]	1.05 [0.77, 1.43]	-	2.06 [0.65, 6.55]
Cesarean Section	1.10 [0.68, 1.79]	0.84 [0.11, 6.67]	1.62 [1.45, 1.80]	1.12 [0.60, 2.09]	0.57 [0.36, 0.91]
Stillbirth	1.31 [0.86, 2.00]	1.10 [0.51, 2.35]	1.17 [0.74, 1.87]	3.83 [1.21, 12.12]	-
Preterm delivery	1.73 [1.30, 2.30]	1.30 [0.38, 4.45]	1.76 [1.39, 2.23]	1.89 [0.48, 7.51]	0.77 [0.39, 1.51]
SGA	2.16 [0.86, 5.46]	1.65 [0.73, 3.74]	5.93 [1.23, 28.55]	-	1.12 [0.08, 14.92]
NICU admission	26.74 [2.36, 303.41]	5.42 [0.12, 250.77]	49.60 [5.08, 484.28]	124.80 [14.26, 1092.24]	-

Abbreviations: AF, atrial fibrillation; AFl, atrial flutter; aRR, multivariable-adjusted Risk Ratio; CI, Confidence Interval; NICU, neonatal intensive care unit; PVCs, premature ventricular contractions; SGA, small for gestational age; SVT, supraventricular tachycardia; VF, ventricular fibrillation; VT, ventricular tachycardia.

## Data Availability

Not applicable.

## Supplementary Materials

The following supporting information can be downloaded at: https://www.mdpi.com/article/10.3390/healthcare14080993/s1, Supplementary Figure S1. Forest Plot of the Association Between Maternal Cardiac Arrhythmias and Preeclampsia, Using Adjusted Estimates; Supplementary Figure S2. Forest Plot of the Subgroup Analysis by Underlying Cardiac Disease for the Outcome of Preeclampsia; Supplementary Figure S3. Forest Plot of the Association Between Maternal Cardiac Arrhythmias and Hypertensive Disorder of Pregnancy, Using Adjusted Estimates; Supplementary Figure S4. Forest Plot of the Association Between Maternal Cardiac Arrhythmias and Gestational Diabetes Mellitus, Using Adjusted Estimates; Supplementary Figure S5. Forest Plot of the Association Between Maternal Cardiac Arrhythmias and Cesarean Section, Using Adjusted Estimates; Supplementary Figure S6. Forest Plot of the Subgroup Analysis by Underlying Cardiac Disease for the Outcome of Cesarean Section; Supplementary Figure S7. Forest Plot of the Association Between Maternal Cardiac Arrhythmias and Stillbirth, Using Adjusted Estimates; Supplementary Figure S8. Forest Plot of the Subgroup Analysis by Underlying Cardiac Disease for the Outcome of Stillbirth; Supplementary Figure S9. Forest Plot of the Association Between Maternal Cardiac Arrhythmias and Preterm Delivery, Using Adjusted Estimates; Supplementary Figure S10. Forest Plot of the Association Between Maternal Cardiac Arrhythmias and Small for Gestational Age Neonates, Using Adjusted Estimates; Supplementary Figure S11. Forest Plot of the Association Between Maternal Cardiac Arrhythmias and NICU Admission, Using Adjusted Estimates; Supplementary Figure S12. Forest Plot of the Subgroup Analysis by Underlying Cardiac Disease for the Outcome of Stillbirth; Supplementary Figure S13. Forest Plot of the Association Between Maternal Cardiac Arrhythmias and Preterm Delivery, Using Adjusted Estimates; Supplementary Figure S14. Forest Plot of the Association Between Maternal Cardiac Arrhythmias and Small for Gestational Age Neonates, Using Adjusted Estimates; Supplementary Figure S15. Forest Plot of the Association Between Maternal Cardiac Arrhythmias and Neonatal Death; Supplementary Figure S16. Forest Plot of the Association Between Maternal Cardiac Arrhythmias and 5-Minute Apgar Score < 7; Supplementary Figure S17. Forest Plot of the Association Between Maternal Cardiac Arrhythmias and NICU Admission; Supplementary Figure S18. Forest Plot of the Association Between Maternal Cardiac Arrhythmias and NICU Admission, Using Adjusted Estimates; Supplementary Table S1. The rest of the characteristics of the included studies; Supplementary Document S1. Search algorithm; Supplementary Document S2. PRISMA checklist. Ref. [48] was cited in the Supplementary Materials.

## Abbreviations

The following abbreviations are used in this manuscript:

aRR	Adjusted risk ratio
CI	Confidence interval
NICU	Neonatal intensive care unit
NOS	Newcastle–Ottawa Scale
PRISMA	Preferred Reporting Items for Systematic Reviews and Meta-Analyses
PVC	Premature ventricular contraction
QUIPS	Quality In Prognosis Studies
RR	Risk ratio
SGA	Small for gestational age
SVT	Supraventricular tachycardia
US	United States
VT	Ventricular tachycardia
