# Supplementary material for: Association of Maternal Cardiac Arrhythmias with Pregnancy Outcomes: A Systematic Review and Meta-Analysis
Source: Healthcare (Basel). 2026 Apr 9;14(8):993. doi: 10.3390/healthcare14080993 (PMC13115657; doi:10.3390/healthcare14080993)
Supplement: Supplementary file 1 [file healthcare-14-00993-s001.zip › Supplementary Table S1.pdf]

Supplementary Table S1. The rest of the characteristics for the included studies.

**Study**	**Arrhythmia Identification**	**Recurrent or new onset arrhythmia**	**Time/Duration of arrhythmia occurence**	**Antiarrhythmics**	**Anticoagulants**	**Cathter Ablation**	**Adequacy of Rate/ Rhythm Control**	**Comorbidities**	**Mitral Valve Disease**	**Congenital or other Structural Heart Disease**	**Definition of Preterm Delivery**	**Small for Gestational Age definition**
Banhidy et al, 2015	Prospective medical logbooks; clinical diagnosis	Differentiated: 45.6% of women had chronic (pre-existing) PSVT; 54.4% had new-onset PSVT during pregnancy.	Highest onset in 2nd–5th gestational months	Antiarrhythmics used by the PSVT group (N=252) included: metoprolol (11.1%), oxprenolol (16.7%), pindolol (6.3%), propranolol (8.7%), and verapamil (6.3%).	Not mentioned.	Not mentioned.	Not mentioned	Pregnant women with an underlying heart disease or essential hypertension were excluded from the analysis.	Excluded	Excluded (idiopathic PSVT only)	<37 weeks of gestation	Not applicable
Bekiaridou et al, 2024	ICD-10 + manual adjudication (ECG, Holter, or telemetry)	Differentiated: Of 76 women with an SVT event, 58 (76.3%) had a prior diagnosis (recurrent), and 18 (23.7%) were new-onset.	Individual duration not determined	14% of patients had a history of using beta-blockers, CCBs, or digoxin; 5.7% had a history of Class IC/III agents. For acute SVT during pregnancy, adenosine was used in 11 women; beta-blockers and	Not mentioned.	Not performed during pregnancy. Ablation was reported as performed prior to (31 patients) and after (11 patients) pregnancy.	Pre-pregnancy ablation highly effective (OR: 0.09)	Hypertension: 3.9% (3 out of 76) in the SVT group and 3.0% (9 out of 304) in the control group.<br><br>Diabetes Mellitus: 7.9% (6 out of 76) in the SVT patients	Excluded	Excluded	<37 weeks of gestation	Not applicable

				other AADs were also initiated/reinitiated.				and 3.3% (10 out of 304) in controls<br><br>Thyroid Disorder: 10.5% (8 out of 76) in the SVT group and 1.0% (3 out of 304) in controls				
Chang et al, 2017	ICD-9 administrative codes (ED/Hospital visits)	Not differentiated in the main analysis. A subgroup with a prior PSVT history was analyzed for recurrence (8.6% without prior ablation vs 2.2% with prior ablation).	Not mentioned	Not mentioned.	Not mentioned.	Not performed during pregnancy. The study only analyzed the effect of ablation performed prior to pregnancy.	Not mentioned	Mothers who had CHD were excluded from the study. The PSVT group and the reference group had similar scores on the Charlson comorbidity index.	Not mentioned	Excluded (CHD)	<37 weeks of gestation	Not applicable
Chou et al, 2023	24-hr Holter monitoring (burden ≥ 1%)	Defined as a condition of frequent PVCs (burden ≥1% on Holter), implying a recurrent/ongoing	Assessed within 1-year window of delivery	No antiarrhythmic drugs (AADs) were used by the PVC group during pregnancy.	Not mentioned.	Not performed during pregnancy. Three patients underwent ablation	Well-tolerated without medical therapy	Diabetes Mellitus: 11.5% (16 out of 139 patients) in the PVC group, vs. 5.9% (3708	Excluded	Excluded (structurally normal hearts only)	<37 weeks of gestation	<10th percentile for GA

		state rather than a single event.				after delivery.		out of 62,819 patients) in the control group<br><br>Hyperthyroidism: 10.8% (15 out of 139 patients) in the PVC group, vs. 2.4% (1500 out of 62,819 patients) in the control group<br><br>Hypothyroidism: 2.2% (3 out of 139 patients) in the PVC group vs. 0.7% (444 out of 62,819 patients) in the control group<br><br>Hypertension: 2.9% (4 out of 139 patients) in the PVC group vs. 3.1% (1956 out of 62,819 patients) in				

								the control group<br><br>Mean BMI: The mean BMI was comparable between the PVC group (22,5±4,3) and the control group (22,2±3,8)				
Ertekin et al, 2016	ROPAC registry (Prospective/Retrospective ECG)	Documents the onset of VA episodes during pregnancy. Does not differentiate between new-onset vs. recurrent based on pre-pregnancy arrhythmia history.	48% of episodes in 3rd trimester	74% of VA patients used medication during pregnancy; this included beta-blockers (57%), other AADs (12%), and amiodarone (3 patients).	LMWH (anticoagulant) use was documented in one patient.	Not mentioned.	Not mentioned	Hypertension: 4.9% in VA group vs. 6.5% in the control group	5 pts (MR/MS)	48% with CHD (Eisenmenger, ToF, ASD, etc.)	<37 weeks of gestation	Birthweight <2,500 grams
Furman et al, 2025	ICD-10 codes (National Inpatient Sample)	Not mentioned.	Not mentioned	Not mentioned.	Not mentioned.	Performed in 190 of 30,215 (0.63%) women during their hospitalization for SVT.	Not mentioned	Obesity: 16.1% in the SVT group vs. 10.5% in the control group<br><br>Hypertension: 5.9% in the SVT group vs. 1.6% in	VHD in 4.7% (type unspecified)	Not mentioned	Not applicable	Not applicable

								the control group<br><br>DM: 3.4% of the SVT group vs. 1.4% in the control group<br><br>Hypothyroidism: 5.9% in the SVT group vs. 3.8% in the control group<br><br>CHF: 4.5% in the SVT group vs. 0.16% in the control group<br><br>VHD: 4.7% of the SVT group versus 0.24% of controls.<br><br>AF: 3.5% of the SVT group compared to 0.05% in controls				

								Previous MI: in 0.36% of the SVT group versus 0.03% of controls				
Henry et al, 2016	Multidisciplinary records + ECG/Echo/Telemetry	Not mentioned.	Not mentioned	Of 36 women with an arrhythmia, 17 (47%) received medication, including 15 who were treated with beta-blockers.	Not mentioned.	Performed in 8 of 36 women (22%) "during or prior to the pregnancy." The specific number performed during pregnancy is not specified.	Not mentioned	Chronic hypertension: 8% in the arrhythmia group vs. 12% in the no arrhythmia group Pre-gestational diabetes mellitus (0% vs. 3%) and gestational diabetes mellitus (6% vs. 7%) were similar between the arrhythmia and no arrhythmia cohorts.<br><br>Women with an arrhythmia were less likely to have a	Present (most frequent non-congenital lesion)	44% Structural Heart Disease; 31% CHD	<37 weeks of gestation	<10th percentile for GA

								congenital cardiac defect (31% vs. 70%), or a structural lesion (44% vs. 89%).				
Hodes et al, 2016	Medical records + ICD interrogation	Differentiated: Sustained VA occurred in 5 of 39 pregnancies (13%). All 5 events were new-onset VAs in women who had no prior history of sustained VA.	Not mentioned	Medications used during 39 pregnancies included beta-blockers (n=16), flecainide (n=1), sotalol, digoxin, and diuretics (n=3).	Not mentioned.	Not mentioned.	82% completed without major events; BB/Flecainide effective	Heart Failure Group: Major Structural RV Disease: Both pregnancies in the HF group (100%) had major structural RV abnormalities pre-pregnancy LV Dysfunction: One of the two pregnancies (50%) in this group began in the setting of LV ejection fraction <55	Not mentioned	100% ARVD/C; 30% with major RV abnormalities	<37 weeks of gestation	Not applicable

								Prior Arrhythmia: One of the two pregnancies (50%) had a prior history of sustained VA/ICD shock.<br><br><u>Ventricular Arrhythmia Group:</u><br><br>Major Structural RV Disease: Only 20% had major structural RV abnormalities before pregnancy<br><br>LV Dysfunction: None of the pregnancies began with LV dysfunction.<br>Prior Arrhythmia: None of the VA events occurred in				

								women with a pre-pregnancy history of sustained VA/ICD shock.<br><br>No Major Events Group:<br><br>Major Structural RV Disease: 27% of pregnancies were begun with major structural RV abnormalities.<br><br>LV Dysfunction: Only one pregnancy (3%) began with LV dysfunction.<br><br>Prior Arrhythmia: 25% of these pregnancies had a pre-pregnancy				

								history of sustained VA/ICD shock.				
Keepanasseril et al, 2024	Hospital records/database (clinical recording)	Differentiated: 47.9% of cases were new-onset AF during pregnancy. Among 36 women with pre-	Mean GA at diagnosis: 24.6 weeks	Most patients were on beta-blockers (73%) and digoxin (54.5%) at their first visit. All were on beta-blockers after an	Two-thirds of the AF group received anticoagulation (heparins, warfarin).	Not mentioned.	Rate-control favored; 31 pts converted to NSR	Pre-gestational DM: 0% (0 out of 71 patients) in the AF group vs. 0.6% (8 out of 1322	46.5% Mod-Severe MS	Excluded (focused on Rheumatic Heart Disease)	<37 weeks of gestation	<10th percentile for GA

		existing AF, 13 had a recurrence.		AF episode, with some requiring verapamil.				patients) in controls<br><br>Hypothyroidism: 10.0% in the AF group and 10.1% in controls<br><br>Hyperthyroidism: 0% in the AF group, compared to 0.7% in controls<br><br>History of HF: 16.9% in the AF group vs. 2.3% in controls				
Lee et al, 2016	ICD-9 + manual EMR/ECG review	Differentiated: 112 of 157 pregnancies were in women with a pre-existing AF diagnosis. 45 cases were new-onset. 15 of the 112 with pre-existing AF had a significant recurrence.	Peak events in 3rd trimester (24h of delivery)	Outpatient prescriptions included beta-blockers (13.4%), CCBs (10.2%), digoxin (5.7%), and sotalol (1.3%). For acute episodes, ibutilide and procainamide were also used.	Anticoagulants LMWH/heparin (3.2%) and aspirin (2.5%) were used.	Not mentioned.	75% converted via meds; 11.7% spontaneous	Pre-existing hypertension: 7.0% in the AF/AFl group compared to 2.9% in the control group<br><br>Hyperlipidemia: 12.4% in the AF group vs. 2.1% in the	~2% RHD	5.4% Structural Heart Disease (CHD/Cardiomyopathy)	<37 weeks of gestation	Not applicable

								control group<br><br>Pre-existing diabetes: 7.0% in the AF group compared to 2.2% in the control group				
Lee et al, 2023	ICD-10 codes (Insurance claims database)	Not mentioned.	Not mentioned	Not mentioned.	Not mentioned.	Not mentioned.	Not mentioned	Not mentioned.	Not mentione d	Acyanotic (292) and Cyanotic (42) CHD; Cardiomy opathy (94)	<37 weeks of gestation	Not applicabl e
Makino et al, 2012	Serial clinical assessments (ECG)	Differentiated: The study included a group with pre-existing arrhythmia diagnoses (n=186) and also identified 7 patients who developed newly documented tachyarrhythmias during pregnancy.	Recorded in logs but not quantified	In the primary arrhythmia group (n=186), one patient with WPW was on digoxin.	In the primary arrhythmia group (n=186), one patient with WPW was on warfarin.	Not mentioned.	Not mentioned	Not mentioned.	MR/MVP (49); MS (2)	295 cases of CHD (VSD, ToF, ASD, etc.)	<37 weeks of gestation	Not applicabl e

Salam et al, 2015	International Registry + ECG/Echo	Differentiated: Of 17 patients with AF/AFL, 7 had a history of the arrhythmia before pregnancy (recurrent), and 10 were new-onset.	Concentrated in 2nd trimester (weeks 23–26)	82% of AF/AFL patients were on medication, including sotalol, digoxin, atenolol, metoprolol, and amiodarone.	The majority of patients received anticoagulation, including LMWH, UH, OAC, and aspirin.	Not mentioned. The authors note that ablation for AF/AFL during pregnancy had not been reported at the time of the study.	Not mentioned	Hypertension: 0% in the AF/AFL group vs. 6.7% (87 out of 1,304) in the control group<br><br>HF: 18% (3 out of 17) in the AF/AFL group vs. 10% (130 out of 1,304) in the control group<br><br>AF/AFL before pregnancy: 11.8% (2 out of 17) in the AF/AFL group vs. 1.8% (23 out of 1,304) in the control group	MS/MR (Major risk factor; OR 6.9)	100% Structural Heart Disease (CHD/VHD/IHD)	<37 weeks of gestation	Not defined
Sharma et al, 2022	EMR query + manual adjudication (ECG/Telemetry)	Differentiated: 45.3% of arrhythmias were recurrent, while 54.7% were newly diagnosed. A breakdown is	SVT/AF: >30s; VA: >3 beats	AADs (beta-blockers, digoxin, sotalol, adenosine, CCBs) were used for maintenance	Anticoagulation was used for acute management in 15 patients with AF.	Not performed during pregnancy. Ablations performed prior to	Not mentioned	Hypertension: 11.0% in the arrhythmia group vs. 4.0% in the	Excluded	Excluded	<37 weeks of gestation	Not applicable

		provided for SVT, AF, and VA subtypes.		and/or acute management.		pregnancy were reported for several patients.		control group<br><br>Diabetes: 4.4% in the arrhythmia group vs. 1.1% in the control group				
Thakkar et al, 2022	ICD-9/10 codes (National Inpatient Sample)	Not mentioned.	Not mentioned	Not mentioned.	Not mentioned.	Not mentioned.	Not mentioned	Obesity: 16.95% in the arrhythmia group vs. 8.31% in the control group<br><br>Hypertension: 6.91% in the arrhythmia group vs. 1.54% in the control group<br><br>Type 2 DM: 1.39% in the arrhythmia group vs. 0.64% in the control group<br><br>Hypothyroidism: 4.02% in the	4.09% VHD (type unspecified)	Not mentioned	Not defined	Not applicable

								arrhythmia group vs. 2.06% in the control group<br><br>Hyperlipidemia: 0.35% in the arrhythmia group vs. 0.06% in the control group<br><br>HF: 3.02% in the arrhythmia group vs. 0.04% in the control group<br><br>VHD: 4.09% in the arrhythmia group vs. 0.13% in the control group<br><br>Previous MI: 0.22% in the arrhythmia group vs. 0.01% in the control group				

								PVD: 0.23% in the arrhythmia group vs. 0.01% in the control group.				
Tong et al, 2018	24-hr Holter monitoring	Documents specific arrhythmic events during pregnancy. In the PVC group, 2 had sustained VT. In the SVT group, 38% had an SVT episode.	Not mentioned	In the PVC group, 47% were treated with beta-blockers (metoprolol, bisoprolol, sotalol); two received IV procainamide for VT.	In the SVT group, 2% were on aspirin (anticoagulant).	Not performed during pregnancy. One patient had a pre-pregnancy VT ablation; another had a postpartum VT ablation.	100% success with medical management	Hypertension: 2% in the PVC group vs. 4% in the SVT control group vs. 0% in the normal control group<br><br>DM: 4% in the PVC group vs. 0% in both the SVT and normal control groups<br><br>Hypothyroidism: 7% in the PVC group vs. 2% in the SVT control group vs. 6% in the normal control group	Excluded	Excluded	<37 weeks of gestation	<10th percentile for GA or <2,500 grams

Wang et al, 2024	Routine ECG + 24-hr Holter for symptoms	Differentiated: Of 62 patients who developed SVT during pregnancy, 22 had a prior history of SVT (recurrent), and 40 were new-onset.	Not mentioned	Not mentioned.	Not mentioned.	Not performed during pregnancy. Ablation was performed post-delivery. 11 patients had undergone a pre-pregnancy ablation, yet 4 of them still developed SVT.	Not mentioned	Left Ventricular Systolic Dysfunction: 3.2% in the SVT group vs. 0% in the non-SVT group<br><br>Left Atrial Enlargement: 3.2% in the SVT group vs. 0% in the non-SVT group<br><br>Pericardial Effusion: 3.2% in the SVT group vs. 0% in the non-SVT group<br><br>Tricuspid Regurgitation: 6.5% in the SVT group vs. 0.4% in the non-SVT group<br><br>Hypertrophic Cardiomyopathy: 1.6% in the SVT group vs. 0%	0% in SVT group; 0.8% in non-SVT group	ASD (1.6%), HCM (1.6%), Pre-excitation	<37 weeks of gestation	<10th percentile for GA

								in the non-SVT group<br><br>NYHA Class III-IV: 17.7% in the SVT group vs. 1.2% in the non-SVT group.				
Wilkie et al, 2025	ICD-10 codes (Epic Cosmos database)	Not mentioned.	Not mentioned	31.5% of the arrhythmia group were on an AAD (e.g., beta-blockers).	Not mentioned.	Reported in <10 patients, but the timing (before, during, or after pregnancy) is not specified.	Not mentioned	Not mentioned.	Not mentioned	100% Ebstein's Anomaly	<37 weeks of gestation	Not applicable
Yeo et al, 2025	ICD-10 codes (Nationwide Readmissions Database)	Not mentioned.	Not mentioned	Not mentioned.	Not mentioned.	Not mentioned. However, cardioversion was performed for SVT (0.5%) and AF (2.6%), and defibrillation was performed for VA (1.9%).	Not mentioned	Pre-existing hypertension: 8.48% in the arrhythmia group vs. 2.87% in the non-arrhythmia group<br><br>Obesity: 17% in the arrhythmia group vs. 10.3% in the non-	5.28% VHD	2.15% CHD	<37 weeks of gestation	Not applicable

								arrhythmia group<br>Pre-existing diabetes mellitus: 2.13% in the arrhythmia group vs. 1.09% in the non-arrhythmia group<br><br>Hyperlipidemia: 1.21% in the arrhythmia group vs. 0.36% in the non-arrhythmia group<br>Valvular heart disease: 5.28% in the arrhythmia group vs. 0.19% in the non-arrhythmia group<br><br>Congestive heart failure: 2.47% in the arrhythmia group vs.				

								0.04% in the non-arrhythmia group<br><br>Congenital heart disease: 2.15% in the arrhythmia group vs. 0.14% in the non-arrhythmia group<br><br>Pulmonary hypertension: 1.10% in the arrhythmia group vs. 0.03% in the non-arrhythmia group<br><br>Coronary artery disease: 0.88% in the arrhythmia group vs. 0.02% in the non-arrhythmia group				

								Prior stroke/TIA: 0.69% in the arrhythmia group vs. 0.10% in the non-arrhythmia group<br><br>Chronic pulmonary disease: 11.70% in the arrhythmia group vs. 5.81% in the non-arrhythmia group<br><br>Anemia: 3.74% in the arrhythmia group vs. 1.99% in the non-arrhythmia group				

Abbreviations: AADs, antiarrhythmic drugs; AF, atrial fibrillation; AFL, atrial flutter; BMI, body mass index; CCBs, calcium channel blockers; CHD : congenital heart disease; CHF, congestive heart failure; DM, diabetes mellitus; GA, gestational age; HF, heart failure; ICD, implantable cardioverter-defibrillator; IV, intravenous; LMWH, low molecular weight heparin; LV, left ventricular; MI, myocardial infarction; N, number; NYHA, New York Heart Association; OAC, oral anticoagulant; PSVT, paroxysmal supraventricular tachycardia; PVC, premature ventricular contractions; PVD, peripheral vascular disease; RV, right ventricular; SVT, supraventricular tachycardia; TIA,

transient ischemic attack; UH, unfractionated heparin; VA, ventricular arrhythmia; VHD, valvular heart disease; VT, ventricular tachycardia; VTA, ventricular tachyarrhythmia; WPW, Wolff-Parkinson-White syndrome.
