# Supplementary material for: Association of Maternal Cardiac Arrhythmias with Pregnancy Outcomes: A Systematic Review and Meta-Analysis
Source: Healthcare (Basel). 2026 Apr 9;14(8):993. doi: 10.3390/healthcare14080993 (PMC13115657; doi:10.3390/healthcare14080993)

Supplementary Figure S1. Forest Plot of the Association Between Maternal Cardiac Arrhythmias and Preeclampsia, Using Adjusted Estimates.

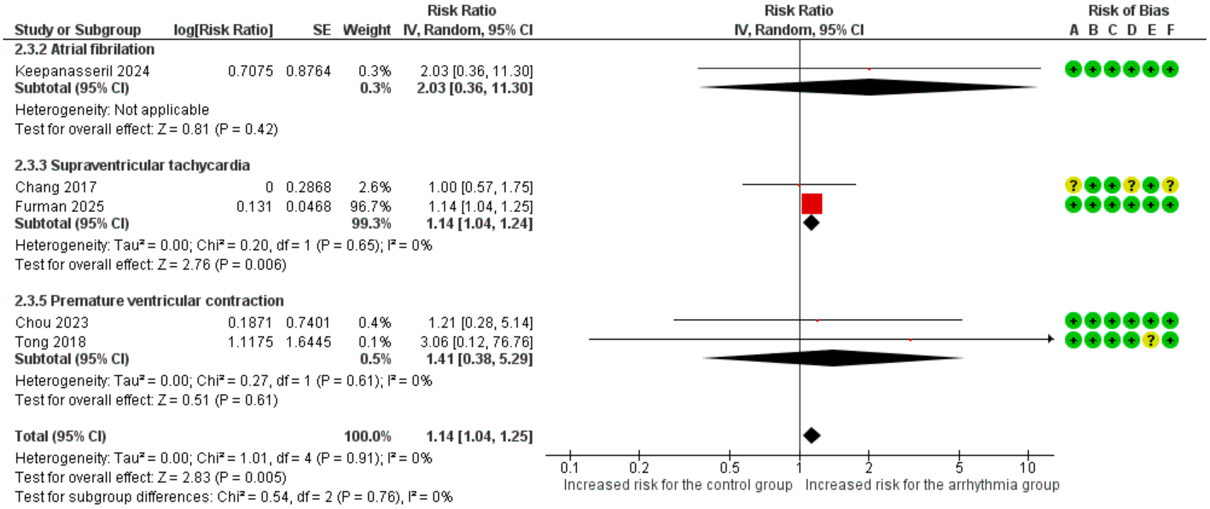

Supplementary Figure S2. Forest Plot of the Subgroup Analysis by Underlying Cardiac Disease for the Outcome of Preeclampsia.

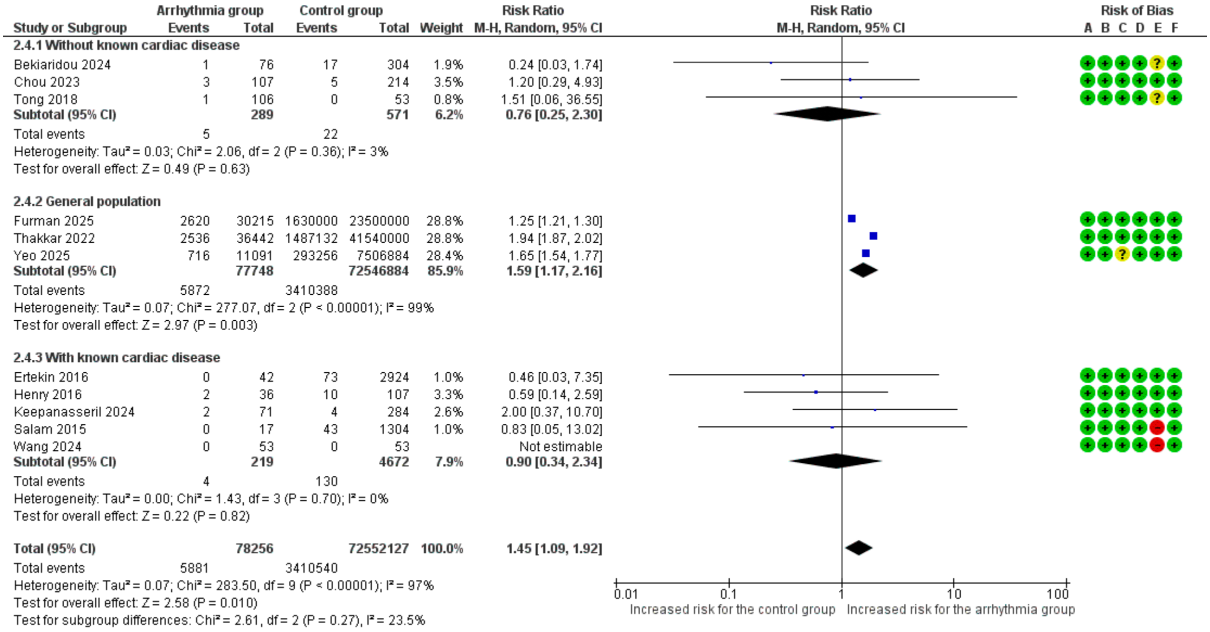

Supplementary Figure S3. Forest Plot of the Association Between Maternal Cardiac Arrhythmias and Hypertensive Disorders of Pregnancy.

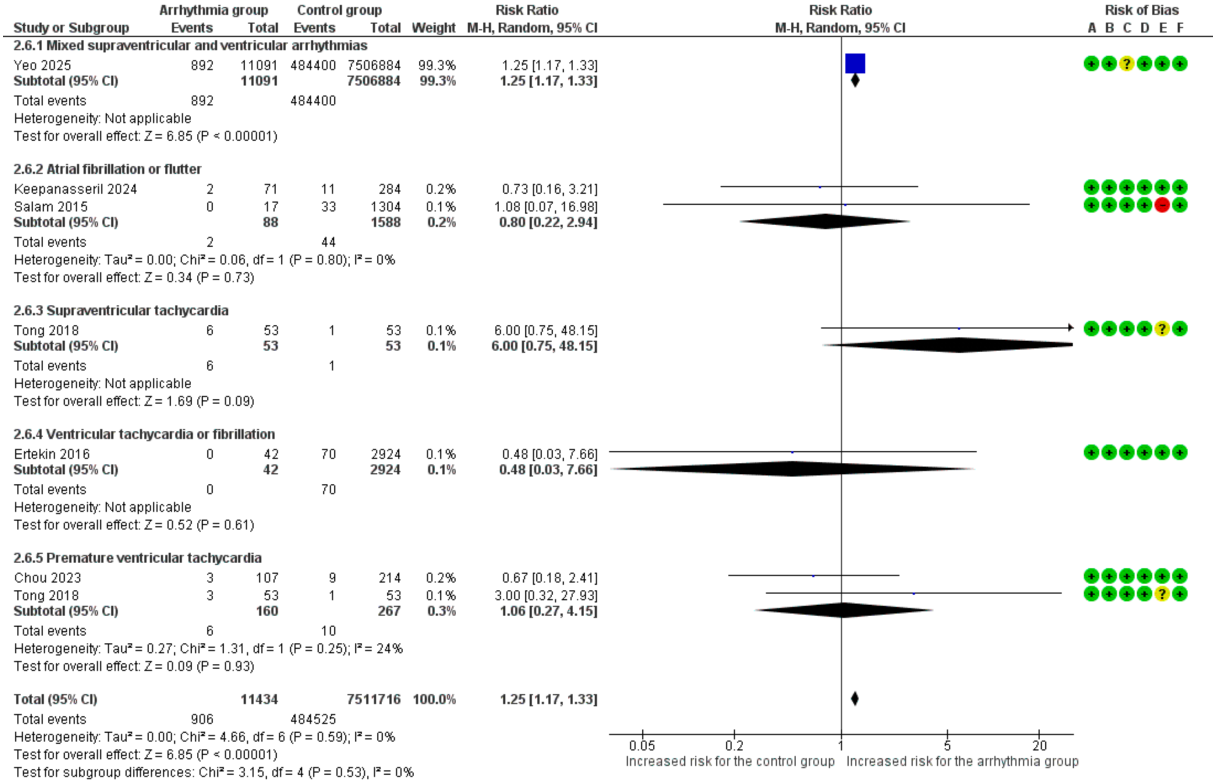

Abbreviations: CI, Confidence Interval; M–H, Mantel–Haenszel method

Supplementary Figure S4. Forest Plot of the Association Between Maternal Cardiac Arrhythmias and Hypertensive Disorder of Pregnancy, Using Adjusted Estimates.

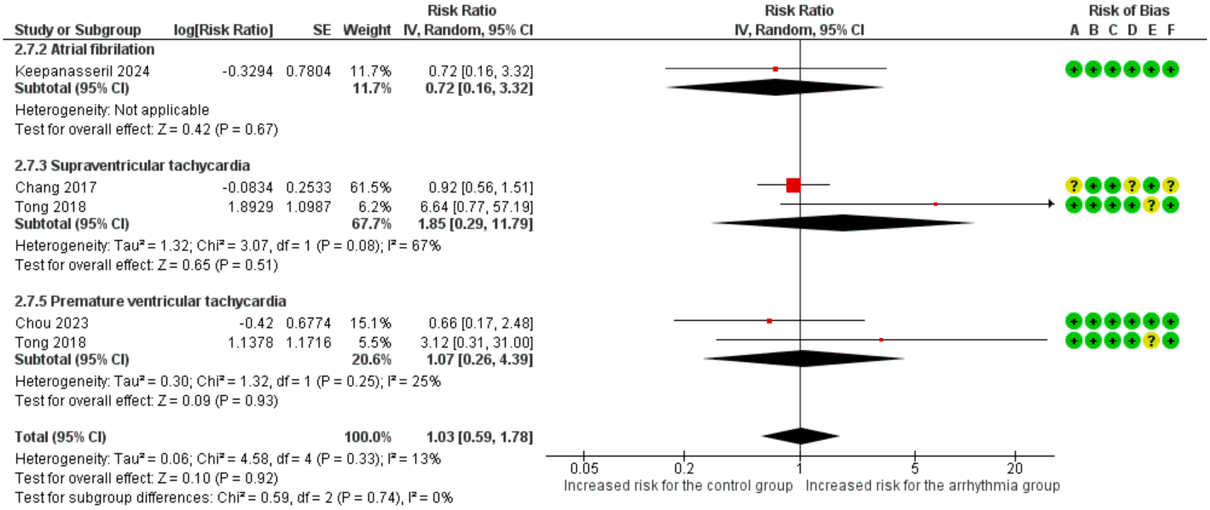

Supplementary Figure S5. Forest Plot of the Association Between Maternal Cardiac Arrhythmias and Gestational Diabetes Mellitus.

Study or Subgroup	Arrhythmia group Events	Arrhythmia group Total	Control group Events	Control group Total	Weight	Risk Ratio M-H, Random, 95% CI	Risk of Bias A B C D E F
**2.9.1 Mixed supraventricular and ventricular arrhythmias**							
Henry 2016	2	36	8	107	5.9%	0.74 [0.17, 3.34]	+ + + + + +
Thakkar 2022	1345	36442	722796	41540000	25.7%	2.12 [2.01, 2.24]	+ + + + + +
Yeo 2025	1090	11091	632396	7506884	25.6%	1.17 [1.10, 1.23]	+ + ? + + +
**Subtotal (95% CI)**		**47569**		**49046991**	**57.2%**	**1.45 [0.83, 2.54]**	
Total events	2437		1355200				
Heterogeneity: Tau² = 0.18; Chi² = 235.33, df = 2 (P < 0.00001); I² = 99%							
Test for overall effect: Z = 1.32 (P = 0.19)							
**2.9.2 Atrial fibrillation or flutter**							
Keepanasseril 2024	7	71	38	284	13.8%	0.74 [0.34, 1.58]	+ + + + + +
**Subtotal (95% CI)**		**71**		**284**	**13.8%**	**0.74 [0.34, 1.58]**	
Total events	7		38				
Heterogeneity: Not applicable							
Test for overall effect: Z = 0.78 (P = 0.43)							
**2.9.3 Supraventricular tachycardia**							
Wang 2024	20	62	44	247	19.8%	1.81 [1.16, 2.84]	+ + + + − +
**Subtotal (95% CI)**		**62**		**247**	**19.8%**	**1.81 [1.16, 2.84]**	
Total events	20		44				
Heterogeneity: Not applicable							
Test for overall effect: Z = 2.59 (P = 0.010)							
**2.9.5 Premature ventricular contraction**							
Chou 2023	6	107	6	214	9.1%	2.00 [0.66, 6.05]	+ + + + + +
**Subtotal (95% CI)**		**107**		**214**	**9.1%**	**2.00 [0.66, 6.05]**	
Total events	6		6				
Heterogeneity: Not applicable							
Test for overall effect: Z = 1.23 (P = 0.22)							
**Total (95% CI)**		**47809**		**49047736**	**100.0%**	**1.42 [0.94, 2.16]**	
Total events	2470		1355288				
Heterogeneity: Tau² = 0.18; Chi² = 239.87, df = 5 (P < 0.00001); I² = 98%							
Test for overall effect: Z = 1.66 (P = 0.10)							
Test for subgroup differences: Chi² = 4.27, df = 3 (P = 0.23), I² = 29.8%							

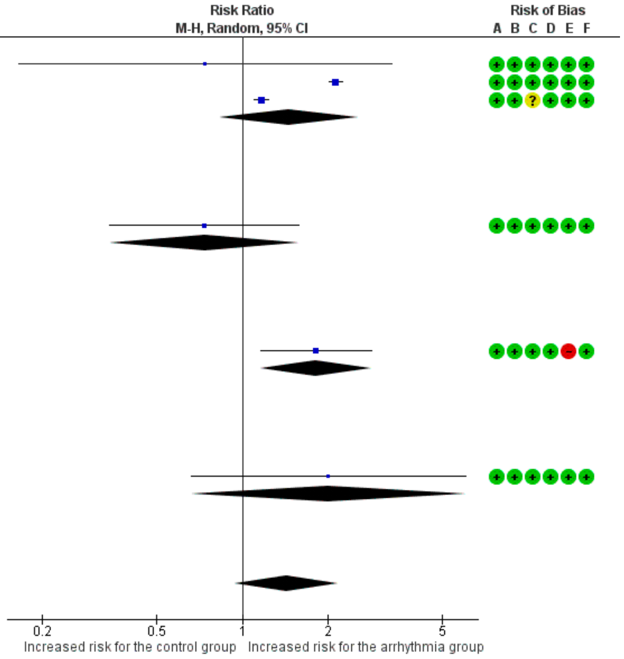

Abbreviations: CI, Confidence Interval; M–H, Mantel–Haenszel method

Supplementary Figure S6. Forest Plot of the Association Between Maternal Cardiac Arrhythmias and Gestational Diabetes Mellitus, Using Adjusted Estimates.

Study or Subgroup	log[Risk Ratio]	SE	Weight	Risk Ratio IV, Random, 95% CI	Risk of Bias A B C D E F
**2.10.2 Atrial fibrilation**					
Keepanasseril 2024	-0.3452	0.4346	13.5%	0.71 [0.30, 1.66]	+ + + + + +
**Subtotal (95% CI)**			**13.5%**	**0.71 [0.30, 1.66]**	
Heterogeneity: Not applicable					
Test for overall effect: Z = 0.79 (P = 0.43)					
**2.10.3 Supraventricular tachycardia**					
Chang 2017	0.0488	0.1582	79.0%	1.05 [0.77, 1.43]	? + + ? + ?
**Subtotal (95% CI)**			**79.0%**	**1.05 [0.77, 1.43]**	
Heterogeneity: Not applicable					
Test for overall effect: Z = 0.31 (P = 0.76)					
**2.10.5 Premature ventricular contraction**					
Chou 2023	0.7224	0.59	7.5%	2.06 [0.65, 6.55]	+ + + + + +
**Subtotal (95% CI)**			**7.5%**	**2.06 [0.65, 6.55]**	
Heterogeneity: Not applicable					
Test for overall effect: Z = 1.22 (P = 0.22)					
**Total (95% CI)**			**100.0%**	**1.05 [0.76, 1.44]**	
Heterogeneity: Tau² = 0.01; Chi² = 2.12, df = 2 (P = 0.35); I² = 6%					
Test for overall effect: Z = 0.28 (P = 0.78)					
Test for subgroup differences: Chi² = 2.12, df = 2 (P = 0.35), I² = 5.9%					

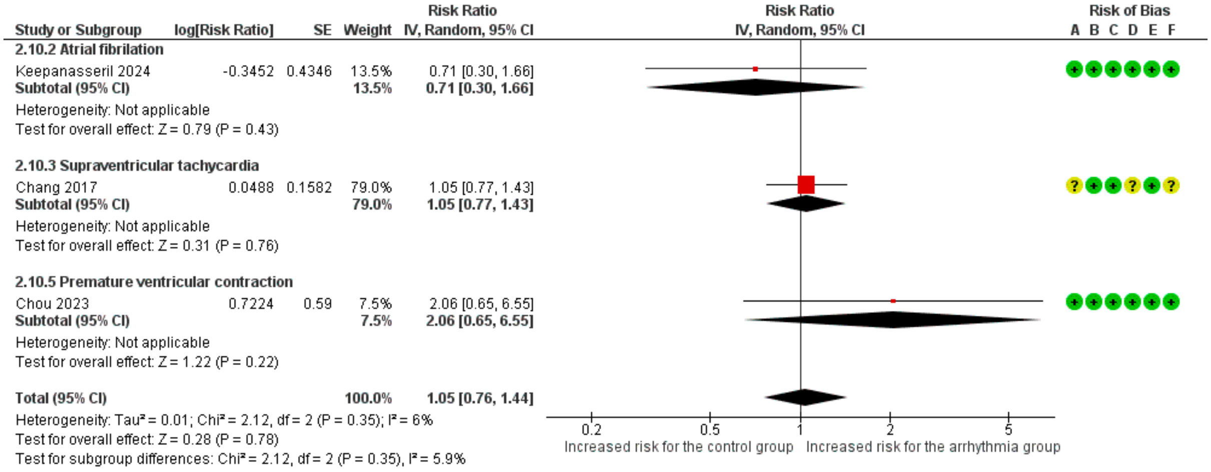

Supplementary Figure S7. Forest Plot of the Association Between Maternal Cardiac Arrhythmias and Cesarean Section.

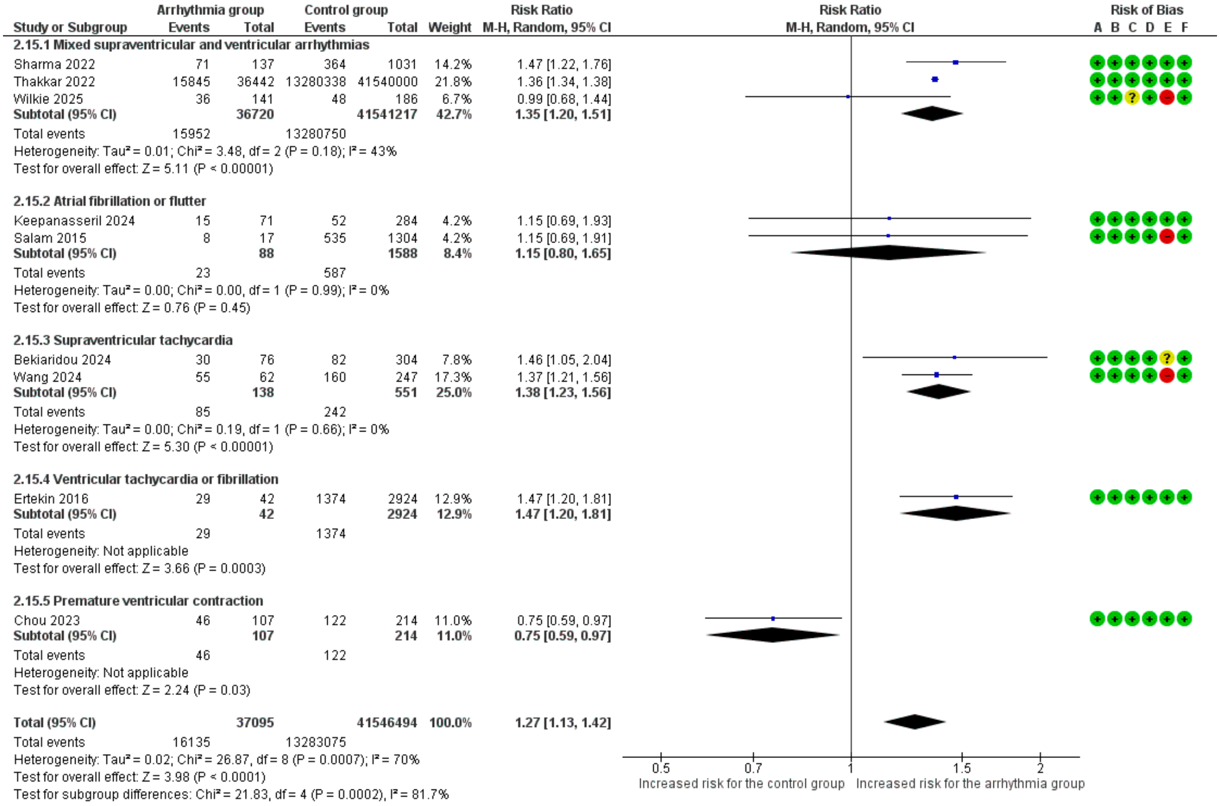

Abbreviations: CI, Confidence Interval; M–H, Mantel–Haenszel method

Supplementary Figure S8. Forest Plot of the Association Between Maternal Cardiac Arrhythmias and Cesarean Section, Using Adjusted Estimates.

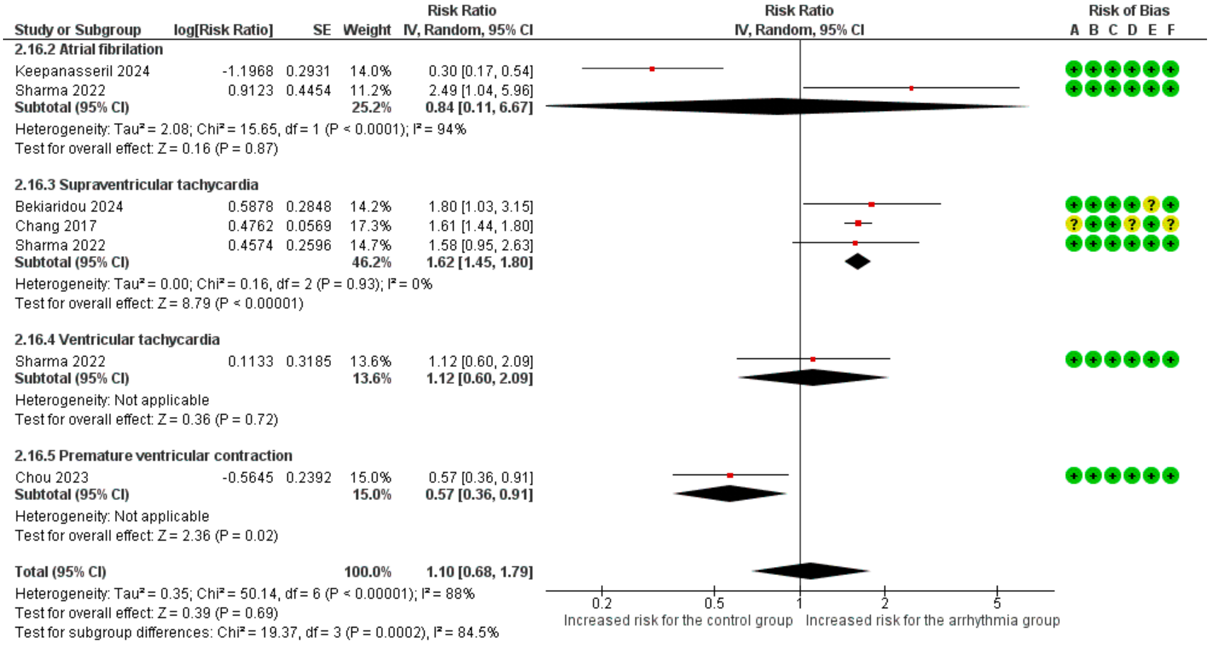

Supplementary Figure S9. Forest Plot of the Subgroup Analysis by Underlying Cardiac Disease for the Outcome of Cesarean Section.

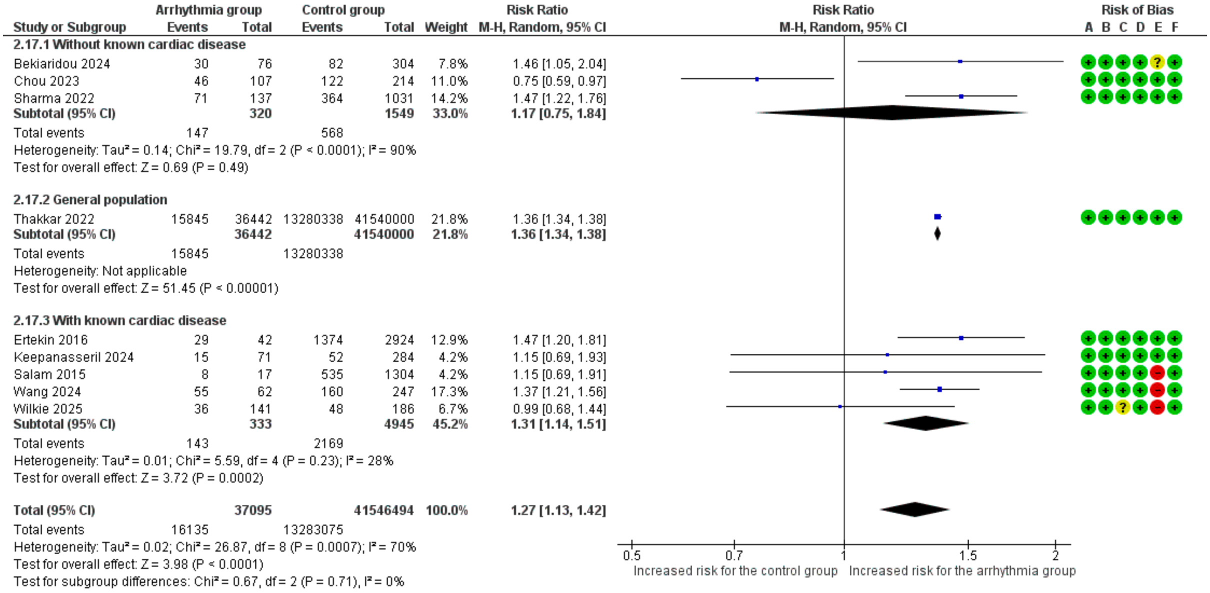

Supplementary Figure S10. Forest Plot of the Association Between Maternal Cardiac Arrhythmias and Placental Abruption.

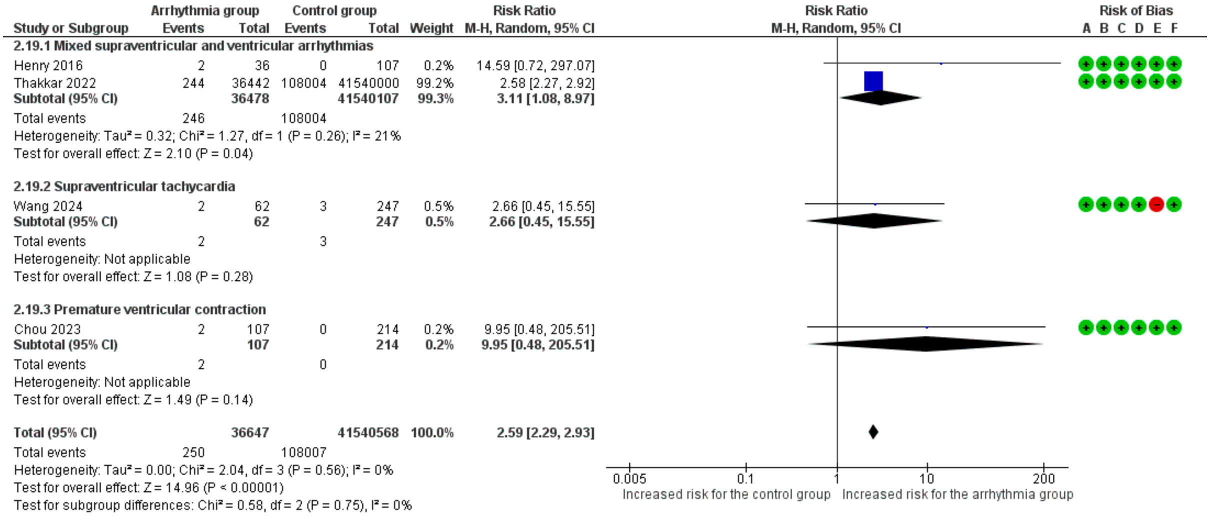

Abbreviations: CI, Confidence Interval; M–H, Mantel–Haenszel method

Supplementary Figure S11. Forest Plot of the Association Between Maternal Cardiac Arrhythmias and Stillbirth, Using Adjusted Estimates.

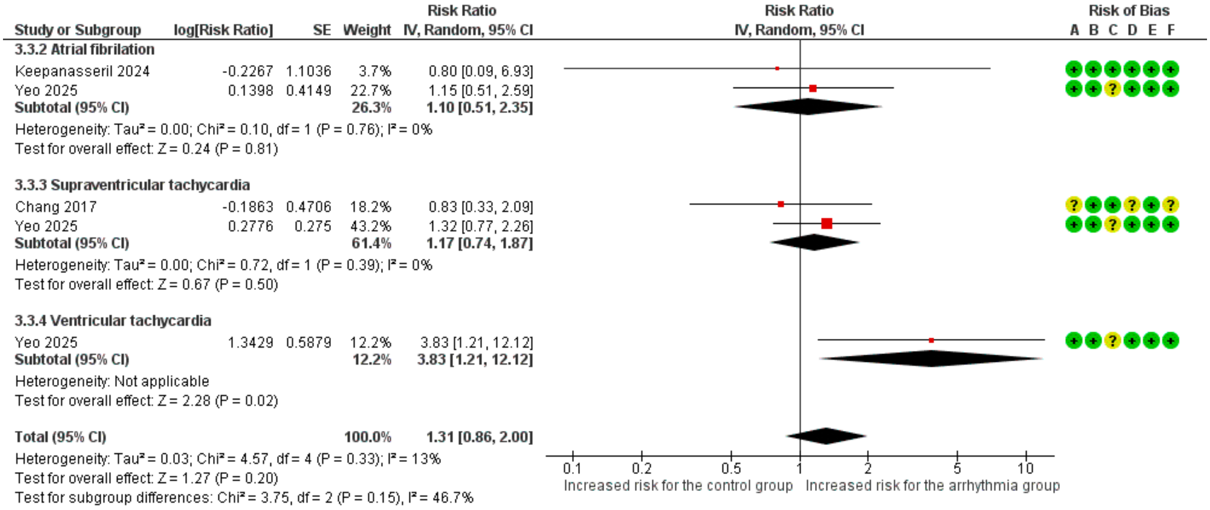

Supplementary Figure S12. Forest Plot of the Subgroup Analysis by Underlying Cardiac Disease for the Outcome of Stillbirth.

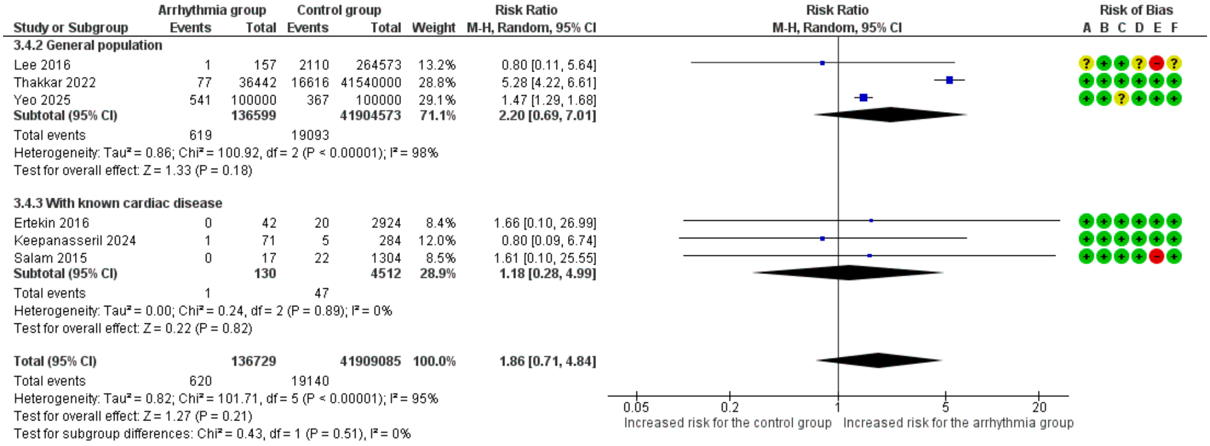

Supplementary Figure S13. Forest Plot of the Association Between Maternal Cardiac Arrhythmias and Preterm Delivery, Using Adjusted Estimates.

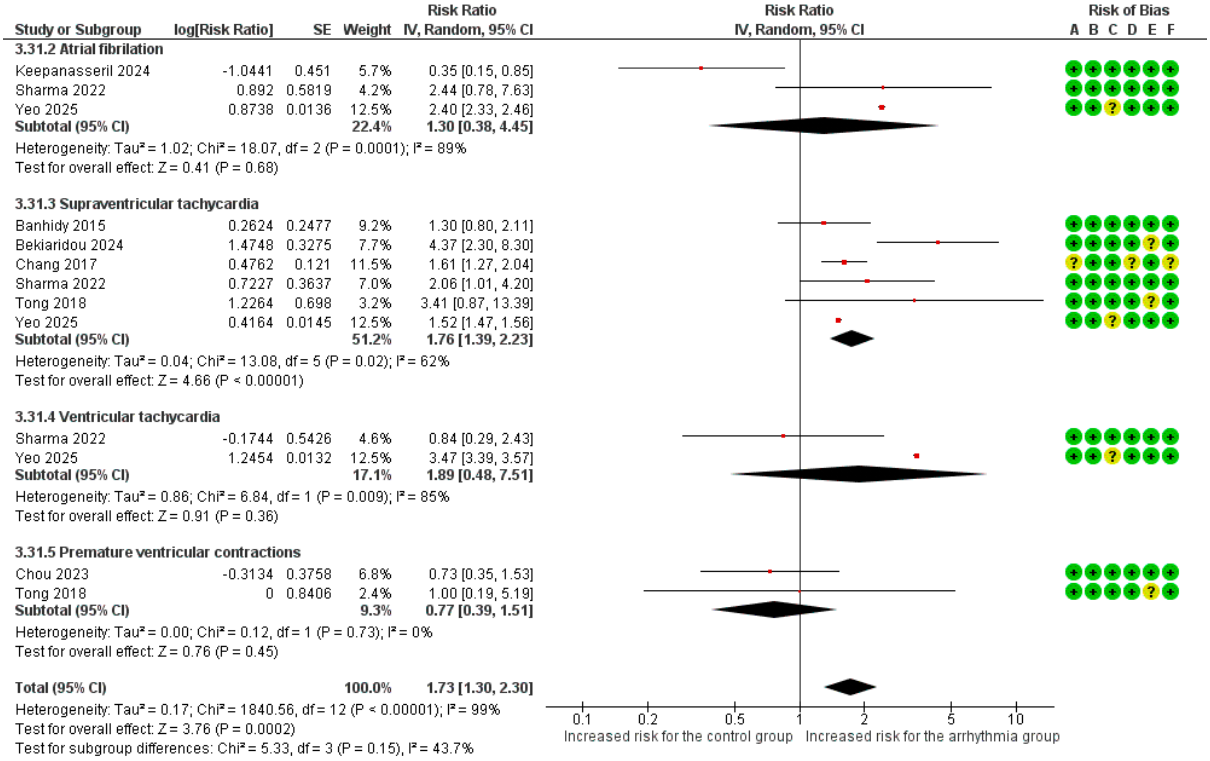

Supplementary Figure S14. Forest Plot of the Association Between Maternal Cardiac Arrhythmias and Small for Gestational Age Neonates, Using Adjusted Estimates.

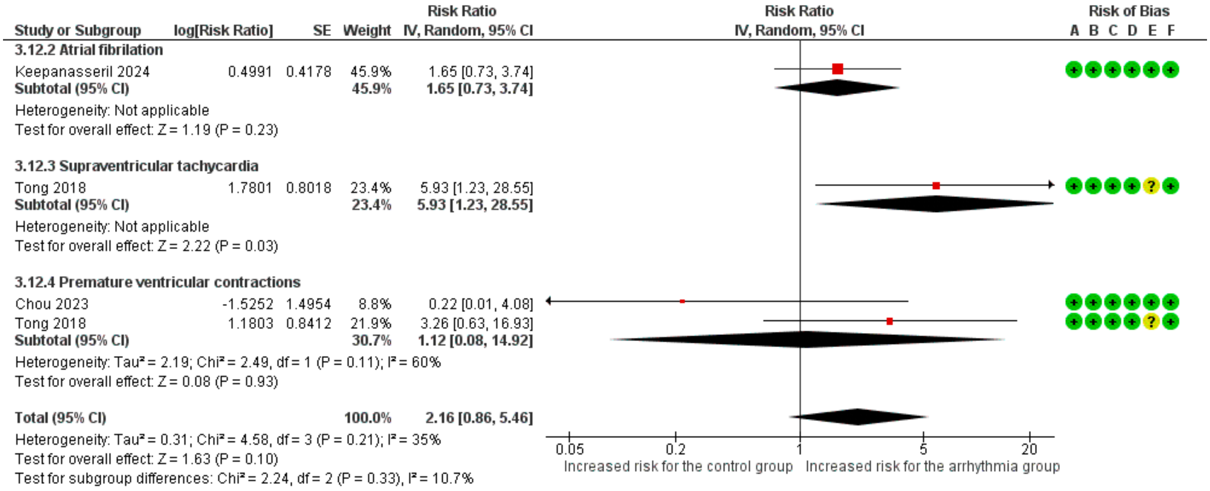

Supplementary Figure S15. Forest Plot of the Association Between Maternal Cardiac Arrhythmias and Neonatal Death.

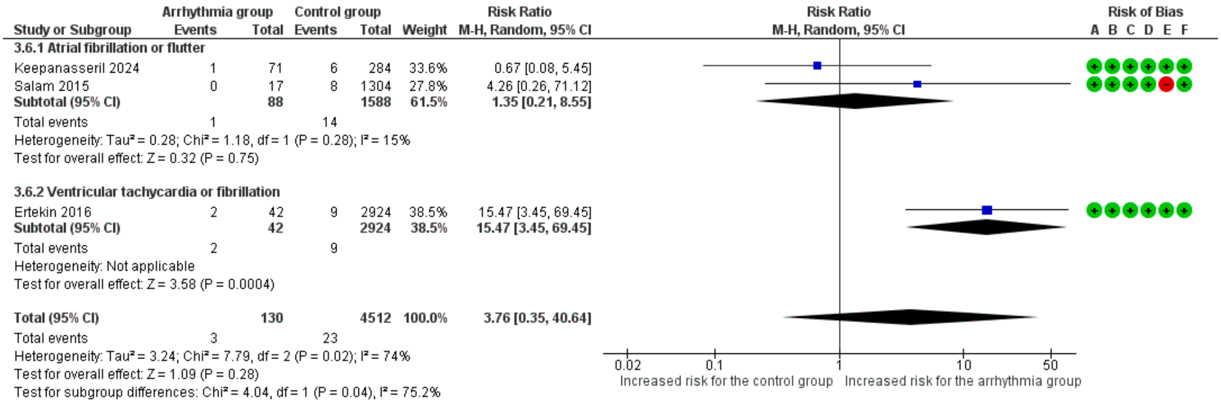

Abbreviations: CI, Confidence Interval; M–H, Mantel–Haenszel method

Supplementary Figure S16. Forest Plot of the Association Between Maternal Cardiac Arrhythmias and 5-Minute Apgar Score < 7.

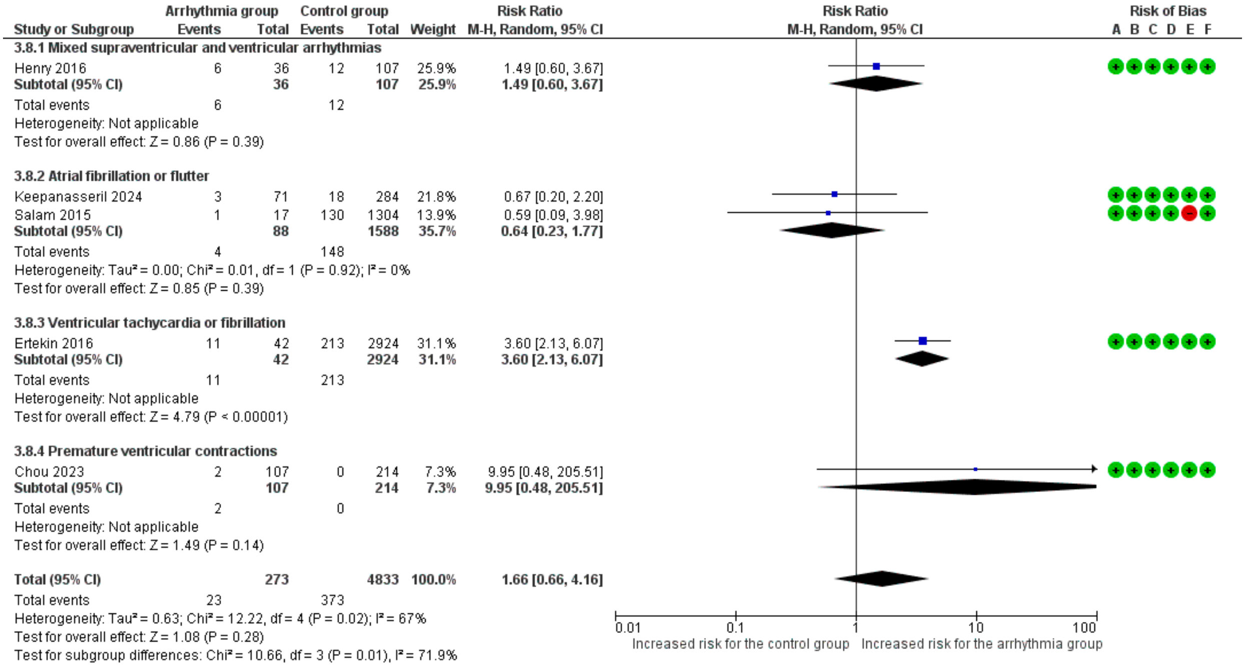

Abbreviations: CI, Confidence Interval; M–H, Mantel–Haenszel method.

Supplementary Figure S17. Forest Plot of the Association Between Maternal Cardiac Arrhythmias and NICU Admission.

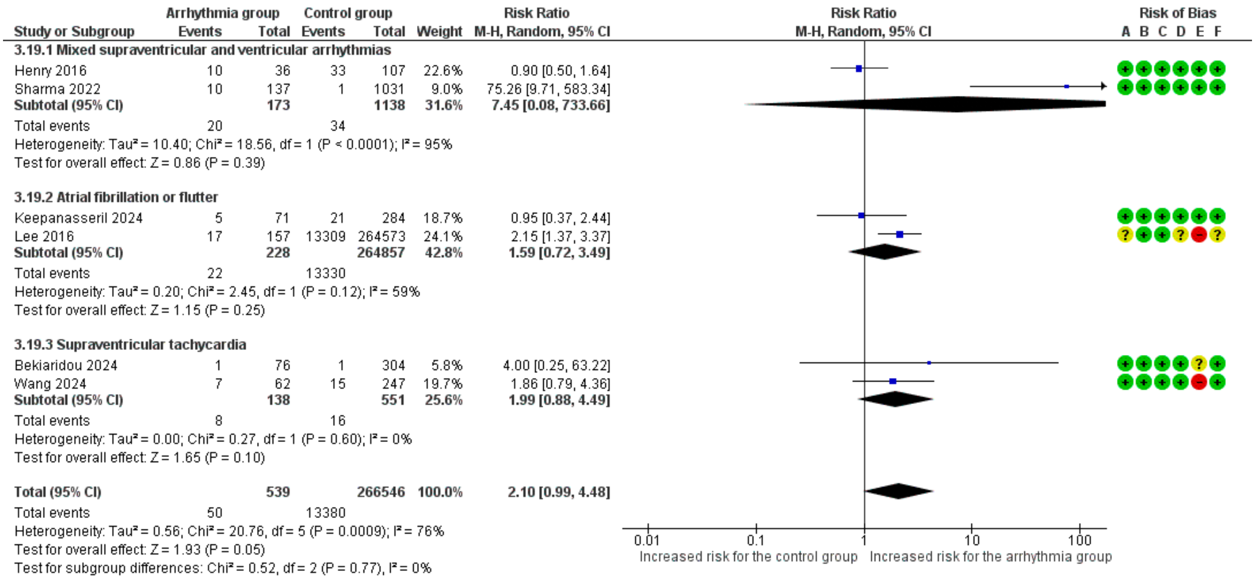

Abbreviations: CI, Confidence Interval; M–H, Mantel–Haenszel method; NICU, Neonatal Intensive Care Unit.

Supplementary Figure S18. Forest Plot of the Association Between Maternal Cardiac Arrhythmias and NICU Admission, Using Adjusted Estimates.

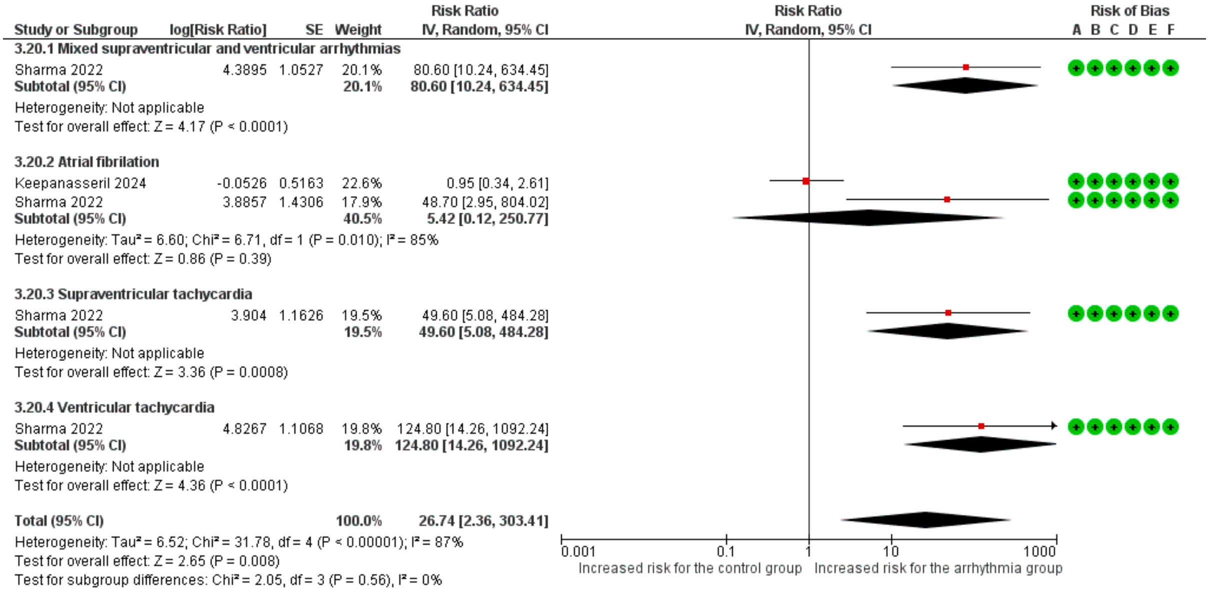

Supplement: Supplementary file 1 [file healthcare-14-00993-s001.zip › Supplementary Figures.pdf]
